# B-cells expressing NgR1 and NgR3 are localized to EAE-induced inflammatory infiltrates and are stimulated by BAFF

**DOI:** 10.1038/s41598-021-82346-6

**Published:** 2021-02-03

**Authors:** Maha M. Bakhuraysah, Paschalis Theotokis, Jae Young Lee, Amani A. Alrehaili, Pei-Mun Aui, William A. Figgett, Michael F. Azari, John-Paul Abou-Afech, Fabienne Mackay, Christopher Siatskas, Frank Alderuccio, Stephen M. Strittmatter, Nikolaos Grigoriadis, Steven Petratos

**Affiliations:** 1 Department of Neuroscience, Central Clinical School, Monash University, Prahran, VIC 3004 Australia; 2 Faculty of Applied Medical Sciences, Taif University, Taif, 26521 Kingdom of Saudi Arabia; 3 Laboratory of Experimental Neurology and Neuroimmunology, Department of Neurology, AHEPA University Hospital, 54636 Thessaloniki, Macedonia Greece; 4 Toolgen Inc., Gasan Digital-Ro, 08594 Geumcheon, Seoul Korea; 5 Department of Microbiology and Immunology, School of Biomedical Science, Peter Doherty Institute for Infection and Immunity, University of Melbourne, Melbourne, VIC 3000 Australia; 6 QIMR Berghofer Medical Research Institute, Herston, QLD 4006 Australia; 7 STEMCELL Technologies, Vancouver, BC V6A 1B6 Canada; 8 Department of Immunology and Pathology, Central Clinical School, Monash University, Prahran, VIC 3004 Australia; 9 Program in Cellular Neuroscience, Neurodegeneration and Repair, Yale University School of Medicine, New Haven, CT 06536 USA

**Keywords:** Neuroscience, Diseases of the nervous system, Neuroimmunology

## Abstract

We have previously reported evidence that Nogo-A activation of Nogo-receptor 1 (NgR1) can drive axonal dystrophy during the neurological progression of experimental autoimmune encephalomyelitis (EAE). However, the B-cell activating factor (BAFF/BlyS) may also be an important ligand of NgR during neuroinflammation. In the current study we define that NgR1 and its homologs may contribute to immune cell signaling during EAE. Meningeal B-cells expressing NgR1 and NgR3 were identified within the lumbosacral spinal cords of *ngr1*^+/+^ EAE-induced mice at clinical score 1. Furthermore, increased secretion of immunoglobulins that bound to central nervous system myelin were shown to be generated from isolated NgR1- and NgR3-expressing B-cells of *ngr1*^+/+^ EAE-induced mice. In vitro BAFF stimulation of NgR1- and NgR3-expressing B cells, directed them into the cell cycle DNA synthesis phase. However, when we antagonized BAFF signaling by co-incubation with recombinant BAFF-R, NgR1-Fc, or NgR3 peptides, the B cells remained in the G0/G1 phase. The data suggest that B cells express NgR1 and NgR3 during EAE, being localized to infiltrates of the meninges and that their regulation is governed by BAFF signaling.

## Introduction

Multiple sclerosis (MS) is a neuroinflammatory disease of the spinal cord, brain and optic nerve manifesting as demyelination and progressive neurodegeneration^1^. In recent years, a number of vital insights into the immunopathogenesis of MS have provided considerable scope for the development of novel therapeutics to be formulated. Mechanisms involving immune cell adhesion and extravasation into the central nervous system (CNS) have, for instance, led to the development of a humanized monoclonal antibody against the cell adhesion molecule α4-integrin, known as natalizumab. Other immunomodulatory drugs have included interferon-β, glatiramer acetate, dimethyl fumarate, alemtuzumab and fingolimod^2^. These treatments are used to diminish the patient’s relapses both in frequency and severity^2^. Recently ocrelizumab has shown promise in the biological’s ability to reduce the relapse rate in MS patients by its ability to specifically target CD20^+^ maturing B-cells that may propagate the disease, with documented potential to limit progression^3–5^. However despite these benefits, chronic-active MS lesions do not respond effectively to the currently available immunomodulatory treatments^6^. This highlights the critical need to develop novel disease modifying agents that can address the gap in treating progressive MS with profound neurodegenerative changes.

Recently, B-cells have emerged as potential important pathogenic players in MS^7–9^. In individuals living with MS, B-cells have been identified to reside within the CNS for protracted periods and are present within the cerebral spinal fluid (CSF). These B-cells may also produce pathogenic autoantibodies intrathecally^10^. In addition, B-cells can be competent (auto) antigen-presenting (APC) cells to effector pathogenic T-cells. Indeed, animal models in which B-cells are specifically lacking MHC-II are resistant to experimental autoimmune encephalitis (EAE), suggesting that the antigen presenting cell (APC) function of B-cells is a key pathogenic mechanism governing disease^11^. B-cells also produce IL-6, a cytokine participating in the emergence of pathogenic Th17 T-cells in MS^12^. In fact, B-cells from MS patients have been demonstrated to produce elevated levels of IL-6^12^. Furthermore, B-cells from MS patients but not from healthy controls produce tumor necrosis factor α (TNFα) along with neurotoxic factors that propagate oligodendrocytopathy^13,14^.

Accumulating evidence thus indicates that blunting the pathogenic effects of B cells could have therapeutic potential in relapsing–remitting multiple sclerosis (RRMS). Development of atacicept, a soluble fusion protein decoy receptor (incorporating the ligand-binding domain of the transmembrane activator and cyclophilin ligand activator (TACI, also known as TNFRSF13B)), designed to block two B-cell cytokines B-cell activating factor of the TNF family (BAFF, also known as BLyS, TNFSF13B) and a proliferation-induced ligand (APRIL, TNFSF13), unexpectedly led to severe adverse events and exacerbated symptoms and lesions when tested in clinical trials in MS patients^15^. The rationale for using this treatment was the observation of elevated BAFF levels in MS lesions and the general knowledge that elevated levels of BAFF can drive autoimmunity^16,17^. This surprising outcome highlighted the complexity of the role of B-cells in MS but also suggests that alternate receptor signaling mechanisms may exist to promote their survival and maturation.

Although the role of BAFF, a known stimulator of B-cell activity^18–20^, has been reported to promote NgR-dependent activation^21^, there seems to be further stratification in the activity related to neuroinflammatory lesions, with antagonists of APRIL/BAFF exacerbating the severity of EAE, an outcome recently reported to relate to APRIL’s anti-inflammatory effects^22^. This deleterious outcome observed during the therapeutic targeting of APRIL has been reported to be a consequence of the ligand’s pleotropic effects in astrocytes, derived from active macrophages during disease progression^22^.

Collectively these findings indicate that APRIL and BAFF are important in regulating autoimmunity^23^. Recent evidence suggests that serum levels of BAFF and APRIL are elevated in MS patients with grey matter neurodegenerative pathology and meningeal inflammation compared with healthy controls^24^. Notably it was reported that these cytokines are reduced in the CSF, during the provision of disease modifying therapies (DMTs)^25^, although surprisingly, they are elevated in the peripheral circulation^26^. Of importance to neurodegenerative changes during injury or disease was the finding that BAFF/BlyS can bind to Nogo Receptor (the high affinity receptor for the myelin associated inhibitory factor, Nogo-A)^21,27,28^, a common receptor expressed in neurons that can limit axonal regrowth and even signal degeneration, in a model of MS^29–31^.

A role for BAFF and BAFF-R in the regulation of primary neurodegeneration without overt inflammatory cell infiltration has been reported but clear mechanistic evidence is still lacking^32^. Both BAFF and BAFF-R are expressed in mouse spinal cord motor neurons and Transgenic SOD-1 mutant mice that were deficient for BAFF-R (*Baffr*^*m/m*^), exhibited increased neurological decline and reduced survival time, implicating neuronal BAFF-R expression as a survival signal for neurons. However, how this relates to neuronal survival signaling during primary neurodegeneration is unclear.

The myelin associated inhibitory factors (MAIFs), consist of Nogo-A, myelin-associated glycoprotein (MAG), and oligodendrocyte-myelin glycoprotein (OMgp) and are expressed on myelin and oligodendrocyte membranes. MAIFs have been demonstrated to be potent inhibitors of axonal regeneration during disease (for review see^33^). Indeed, we previously reported that Nogo-A and its cognate receptor NgR1, may promote axonal degeneration in the animal model of MS, EAE^30,31^. It has also been hypothesized that remyelination failure observed in progressive MS may be a result of co-activation of MAIFs within extracellular myelin debris, acting as potent endogenous inhibitory cues to block the recruitment and maturation of oligodendroglial precursor cells (OPCs) around demyelinated plaques^34–36^. MAIF-dependent axonal outgrowth inhibition is promulgated through activation of the small GTPase RhoA, inducing actin and tubulin disassembly via a signaling pathway which may be kinase-dependent^37–39^. Downstream signaling occurs following high-affinity ligand-mediated tripartite receptor complexing of the NgR1, low-affinity neurotrophin receptor (p75^NTR^), and LINGO-1; all expressed on axons^40–42^. However, how the proposed BAFF/NgR1 interaction can regulate active demyelination of lesions that are pathognomonic for MS, has not been elucidated.

In this study, we show for the first time the effect of BAFF on NgR-expressing B-cells isolated at the peak stage of EAE in the MOG_35-55_-induced mouse model of MS. We found that both NgR1 and NgR3 may play a role in modulating the adaptive immune response during the progression of MOG_35-55_-induced EAE and demonstrate that mature B-cells express NgR3 within spinal cord infiltrates, localized to the meninges during EAE. Our systematic dissection of the immune function of NgR during CNS inflammation has identified that the NgR-expressing B-cells can be stimulated by BAFF to induce proliferation and differentiation that can be antagonized by the provision of Nogo receptor fusion proteins (NgR-Fc and NgR3-P). We further demonstrate that these leptomeningeal NgR-expressing B-cells can actively secrete anti-myelin glycoprotein antibodies that bind to myelin and oligodendrocytes in the mouse spinal cord white matter. Our data highlight the complexity of BAFF signaling during neuroinflammation and suggest that both NgR1 and NgR3 homologs can be important regulators of the neurodegenerative process governing EAE and by extension MS.

## Results

### MOG_35-55_ and rMOG-induction of EAE in *ngr1*^***−/−***^ exhibit reduced clinical severity, demyelination and axonal loss

We have previously reported that deletion of the *ngr1* allele (exon 2) limits the progression of EAE and axonal degeneration^30,31^. In the current set of experiments we have interrogated further the immunological role of NgR1, by immunizing female wildtype (*ngr1*^+*/*+^) and NgR1 deficient (*ngr1*^*−/−*^) mice with either MOG_35-55_ peptide or recombinant MOG(1-125) [rMOG]. The effect of *ngr1* gene deletion on the clinical course in MOG_35-55_ and rMOG-induction of EAE is shown in Table 1 (Table 1a, b), demonstrating a delay in the onset and severity of clinical disease signs. CFA inoculated animals did not develop EAE in any of two cohorts.

**Table 1:** a. Effect of *ngr1* gene deletion on the clinical course in MOG_35-55_ EAE. b. Effect of *ngr1* gene deletion on the clinical course in rMOG EAE.

	*ngr1*^+/+^	*ngr1*^*−*/*−*^	*p* value
**a**			
Incidence	76/99	32/60	
Mean day of disease onset (dDO)	13.5 ± 0.3	16.1 ± 0.6	0.012
Mean maximal score (MMS)	3.2 ± 0.2	2.3 ± 0.1	0.018
Area under the curve (AUC)	39.2 ± 3.1	22.2 ± 2.3	0.001
Mortality rate	3/99	2/60	
**b**			
Incidence	21/22	10/12	
Mean day of disease onset (dDO)	14.1 ± 0.4	20.6 ± 0.9	0.008
Mean maximal score (MMS)	2.9 ± 0.2	2.1 ± 0.2	0.022
Area under the curve (AUC)	33.9 ± 2.5	15.3 ± 1.7	0.001
Mortality rate	1/22	1/12	

^a^Disease parameters of the respective EAE clinical course in *ngr1*^+/+^ and *ngr1*^*−*/*−*^ mice, as shown in Fig. 1a. Data represent mean ± SEM.

^b^Disease parameters of the respective EAE clinical course in *ngr1*^+/+^ and *ngr1*^*−*/*−*^ mice, as shown in Supplementary Fig. 1a. Data represent mean ± SEM.

**Figure 1:**
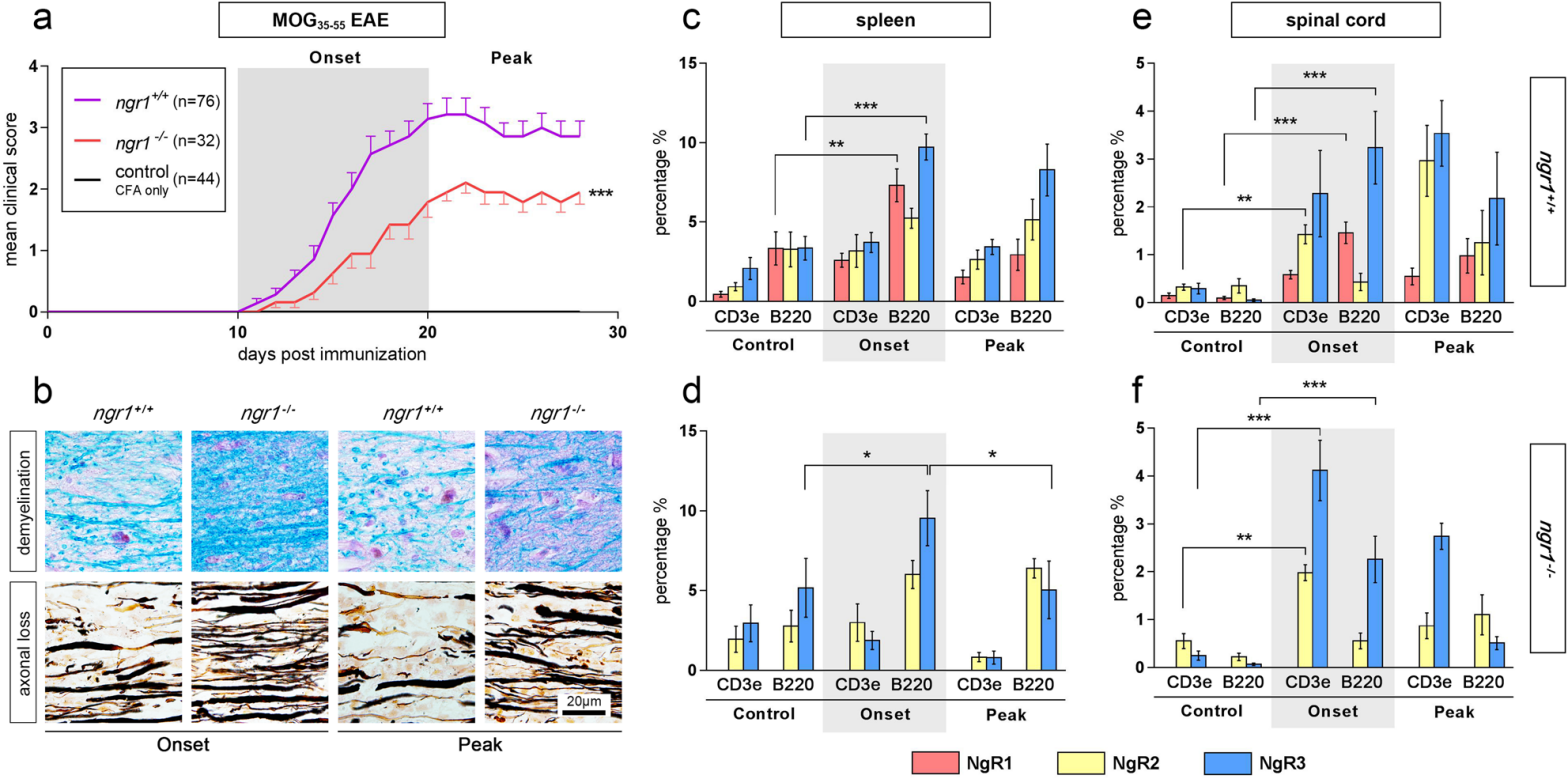
The onset of MOG_35-55_-induced EAE mice is accompanied by an increase in NgR1^+^ and NgR3^+^ immune cell populations. (**a**) There was a significant delay in the onset of EAE and reduction in severity for the *ngr1*^*−/−*^ (n = 32; red line) compared to *ngr1*^+*/*+^ mice (n = 76; purple line) within the 2 main phases studied; onset (days 10–20 post-immunization; 10–20 dpi) and peak (days 20–30 post-immunization; 20–30 dpi). A control group (CFA-injected only; n = 44; black line) was also included. Daily clinical scores represent mean ± SEM, ***p < 0.001 (from day 15 until end-point), two-way ANOVA. (**b**) The severity of demyelination and axonal loss was also reduced in the spinal cord of *ngr1*^*−/−*^ EAE-induced mice, for both onset and peak phases, determined by Luxol fast blue (LFB)/Periodic acid-Schiff (PAS) and Bielschowsky silver stain, respectively. (**c**,**d**) Flow cytometric analysis of double-labeled cell suspensions is from the spleen of both *ngr1*^+*/*+^ and *ngr1*^*−/−*^ mice showed an elevated NgR3 expression in B-cells (B220^+^) at the onset of EAE (n = 5; *t* test ***p < 0.001 and *p < 0.05 compared to controls, respectively) along with a concomitant increase of NgR1 in *ngr1*^+*/*+^ mice (n = 5; *t* test **p < 0.01). B-cells co-expressing NgR3 were subsequently reduced in the peak stage of EAE (n = 5; *t* test *p < 0.05) in *ngr1*^*−/−*^ mice that exhibited disease signs. The NgR2 homolog did not manifest any discernible significant increase in either *ngr1*^+*/*+^ or *ngr1*^*−/−*^ mice. (**e**,**f**) The percentages of double labeled immune cells isolated from the spinal cord in EAE-induced mice were again significantly elevated for NgR3 and NgR1 during the onset of disease, compared to controls (n = 5; *t* test ***p < 0.001 for both). Interestingly, deletion of the *ngr1* gene in *ngr1*^*−/−*^ mice incurred an NgR3 and NgR2 upregulation in T-cell (CD3e^+^) populations (n = 5; *t* test ***p < 0.001 and **p < 0.01 compared to controls, respectively). B220: B-cell marker; CD3e: T-cell marker. Bars represent mean ± SEM, *p < 0.05, **p < 0.01, ***p < 0.001.

Consistent with our previous MOG_35-55_ data, a significant delay in the onset of clinical symptoms (dDO; 16.1 ± 0.6 in *ngr1*^*−/−*^ vs 13.5 ± 0.3 in *ngr1*^+*/*+^, *p* < 0.05) and reduced disease burden (MMS; 2.3 ± 0.1 in *ngr1*^*−/−*^ vs 3.2 ± 0.2 in *ngr1*^+*/*+^, *p* < 0.05 and AUC; 22.2 ± 2.3 in *ngr1*^*−/−*^ vs 39.2 ± 3.1 in *ngr1*^+*/*+^, *p* < 0.001) has been observed in the *ngr1*^*−/−*^ compared to the *ngr1*^+*/*+^ mice (Fig. 1a; Two-way ANOVA, *p* < 0.001). Histopathological evaluation performed with LFB/PAS and Bielschowsky staining, illustrated a significantly reduced demyelination in *ngr1*^*−/−*^ mice at disease onset and reduced axonal loss at the peak stage of disease, in comparison to *ngr1*^+*/*+^ mice (Fig. 1b).

Similar results were demonstrated in the rMOG-inoculated group, a model of EAE-induction commonly utilized to induce overt B-cell activation, differentiation and anti-myelin autoantibodies^43–45^. We again observed a significant delay in EAE onset (dDO; 20.6 ± 0.9 in *ngr1*^*−/−*^ vs 14.1 ± 0.4 in *ngr1*^+*/*+^, *p* < 0.01) and reduced severity (MMS; 2.1 ± 0.2 in *ngr1*^*−/−*^ vs 2.9 ± 0.2 in *ngr1*^+*/*+^, *p* < 0.05 and AUC; 15.3 ± 1.7 in *ngr1*^*−/−*^ vs 33.9 ± 2.5 in *ngr1*^+*/*+^, *p* < 0.001) in the *ngr1*^*−*/*−*^ mice following rMOG-immunization (Additional file 1: Fig. S1a; Two-way ANOVA, *p* < 0.001). Demyelination and axonal loss exhibited a similar reduction in the pattern of degenerative change in the *ngr1*^*−/−*^ mice following rMOG induction as that observed in the MOG_35-55_ EAE model (Additional file 1: Fig. S1b).

### B-cells isolated from spleen and spinal cord of ***ngr1***^+***/***+^ and ***ngr1****−/−* mice following either MOG_35-55_ or rMOG EAE induction express the NgR1 and NgR3 homolog

To interrogate the differing immunopathogenic mechanisms potentially governing the EAE clinical outcomes attributed to *ngr1*^+*/*+^ and *ngr1*^*−/−*^ mice following MOG_35-55_ peptide and rMOG protein, we defined the co-expression profiles of selected immune cells from isolated populations (B220 for B-cells and CD3e for T-cells) with flow cytometry analysis, according to the three NgR homologs namely NgR1 (*Rtn4r*), NgR2 (*Rtn4rl2*) and NgR3 (*Rtn4rl1*) [Fig. 1 and Additional file 2: Fig. S2].

In the spleen of MOG_35-55_ EAE-induced *ngr1*^+*/*+^ mice, B-cells expressing NgR1 and NgR3 were significantly increased upon disease onset (*p* < 0.01 and *p* < 0.001, respectively), but as the disease progressed beyond the peak stage of EAE NgR1^+^ B-cells were markedly reduced while NgR3^+^ B-cells remained elevated at the peak stage of disease (Fig. 1c; and Additional file 2: Fig. S2). In the spleen of *ngr1*^*−/−*^ mice there were elevated numbers of B-cells expressing NgR3 during disease onset (9.5 ± 1.7 vs controls 5.2 ± 1.8, *p* < 0.05), but these numbers were significantly reduced (5.1 ± 1.7, *p* < 0.05) upon progression, at the peak stage of EAE (Fig. 1d; and Additional file 2: Fig. S2).

Interestingly, in the spinal cord of the *ngr1*^+*/*+^ mice, both T- and B-cell populations expressing NgR3 were elevated at the onset of EAE (2.2 ± 0.9 vs controls 0.2 ± 0.1, *p* < 0.05 for CD3e^+^ T-cells and 3.2 ± 0.7 vs controls 0.05 ± 0.02, *p* < 0.05 for B220^+^ B-cells) and remained elevated at the peak stage of disease (Fig. 1e; and Additional file 2: Fig. S2). This elevated NgR3 expression profile in T- and B-cells (*p* < 0.001 vs controls for both populations) was also replicated in the *ngr1*^*−/−*^ mice at the onset of EAE (Fig. 1f; and Additional file 2: Fig. S2). Intriguingly, an upregulation in NgR2^+^ T-cells (*p* < 0.01 vs controls) was also observed in both *ngr1*^+*/*+^ and *ngr1*^*−/−*^ mice.

Thus, deletion of the *ngr1* gene in mice does not result in an altered infiltration of B-cells during disease induction but there are altered numbers of NgR3-expressing cells during the peak stage of EAE that may relate to the reduced severity observed in this genotype following MOG_35-55_ immunization. Collectively, these data indicate that NgR1 and NgR3 are expressed on specific numbers of immune cells particularly of the B cell lineage, within the CNS compartment upon the induction of EAE, and thus both of these receptors may influence the behaviour of these cells once resident in the CNS. This does not seem to be the case with the NgR2 homolog.

Next, we examined the subset of immune cells present in the spleen and spinal cord harvested from *ngr1*^+*/*+^ and *ngr1*^*−/−*^ mice compared to controls, following EAE induction by immunization with rMOG. In the spleen, the percentage of B-cells expressing NgR (NgR1 and 3 in *ngr1*^+*/*+^ and NgR3 in *ngr1*^*−/−*^ specifically) increased significantly (13.8 ± 1.0 vs controls 3.3 ± 1.0, *p* < 0.001 for NgR1 in *ngr1*^+*/*+^and 7.7 ± 1.9 vs controls 5.1 ± 1.8, *p* < 0.01 for NgR3 in *ngr1*^*−/−*^) at the onset of the disease (Additional file 1: Fig. S1c, d; and Additional file 2: Fig. S2).

The B-cells expressing NgR1 in the spinal cord of *ngr1*^+*/*+^ mice were elevated (2.4 ± 0.6 vs controls 0.09 ± 0.03, *p* < 0.001) at the disease onset (Additional file 1: Fig. S1e; and Additional file 2: Fig. S2). Moreover, NgR3^+^ B-cells were significantly elevated in the spinal cord of the *ngr1*^+*/*+^ (3.5 ± 0.6 vs controls 0.05 ± 0.02, *p* < 0.001) and *ngr1*^*−/−*^ mice (1.0 ± 0.2 vs controls 0.06 ± 0.02, *p* < 0.01) (Additional file 1: Fig. S1e, f; and Additional file 2: Fig. S2). Intriguingly, the numbers of NgR3-expressing B-cells resident in the CNS during EAE progression at the peak stage of disease in *ngr1*^*−/−*^ mice were substantially reduced back to basal levels prior to disease onset.

Collectively these results demonstrate that NgR3 is expressed on increased numbers of B-cells within the CNS during the onset of EAE symptoms following either, MOG_35-55_- and rMOG-induction, demonstrated in both *ngr1*^+*/*+^ and *ngr1*^*−/−*^ mice. However, these NgR3 expressing B-cells demonstrated reduced numbers toward basal levels in the CNS during the peak stage of EAE when profound neurodegeneration is evident. The data implicate a potential signaling role for NgR3 in B-cells while in the CNS compartment at the induction of inflammatory disease.

Due to the similarities between the two MOG-induced experimental models with regards to the clinical/immunopathological and the co-expression outcome of the Nogo receptor homologs, we proceeded with the MOG_35-55_ EAE material for the remaining analysis. At the same time, we excluded NgR2 as a valuable parameter to be further investigated, due to the lack of strong evidence of its implication to the disease induction and progression.

### NgR1^+^ and NgR3^+^ B-cells are localized within infiltrates of the spinal cord during the onset of EAE symptoms

**Figure 2:**
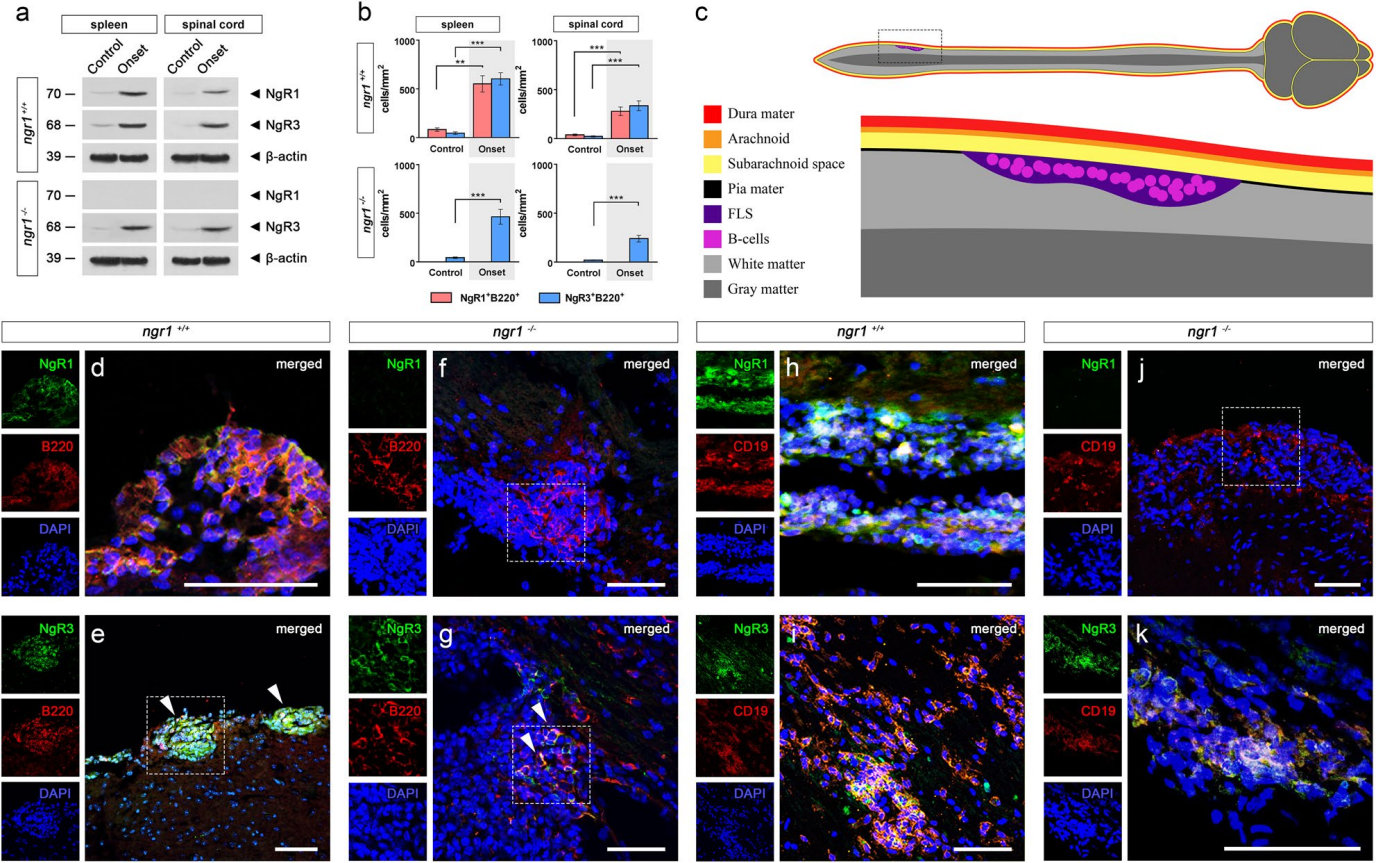
NgR1 and NgR3 are localized to B-cells within lymphoid-like follicles of the spinal cord. (**a**) Western blot of spleen and spinal cord extracts from *ngr1*^+*/*+^ and *ngr1*^*−/−*^ mice showcasing the expression of NgR1 and NgR3. β-actin was used as a loading control. (**b**) Semi-quantitative analysis of both NgR1- and NgR3-positive B-cells per unit area (mm^y^) of spleen and spinal cord infiltrates for the *ngr1*^+*/*+^ and *ngr1*^*−/−*^ mice (n = 34 *ngr1*^+*/*+^ mice; 108 sections, n = 15 *ngr1*^*−/−*^ mice; 40 sections) during the onset of EAE. These B-cells demonstrated a highly inducible receptor expression (student’s *t* test with Bonferroni correction ***p < 0.001 and **p < 0.01) compared to controls. (**c**) Illustration of inflammatory infiltrates analyzed via immunofluorescence immunolabeling of follicle-like structures (FLS) within the lumbosacral spinal cords of EAE-induced mice in (**d**–**k**). (**d**) Representative NgR1 and B220 double-labeled images showing the expression on clusters of B-cells localized to infiltrates around the leptomeninges during the onset of EAE in *ngr1*^+*/*+^ mice. (**e**) Representative NgR3 and B220 double-labeled images showing the expression on clusters of B-cells localized to infiltrates around the leptomeninges during the onset of EAE in *ngr1*^+*/*+^ mice (hatched boxes). (**f**) Representative images for the *ngr1*^*−/−*^ mice, highlighting the absence of the NgR1 protein expression in clusters of B-cells in similar infiltrates of the leptomeninges during the onset of EAE (hatched boxes). (**g**) Representative images for the *ngr1*^*−/−*^ mice, highlighting the strong expression of the NgR3 protein expression in clusters of B-cells in similar infiltrates of the leptomeninges during the onset of EAE (hatched boxes). (**h**) Representative NgR1 and CD19 double-labeled images showing the expression on clusters of B-cells localized to infiltrates around the leptomeninges during the onset of EAE in *ngr1*^+*/*+^ mice. (**i**) Representative NgR3 and CD19 double-labeled images showing the expression on clusters of B-cells localized to infiltrates around the leptomeninges during the onset of EAE in *ngr1*^+*/*+^ mice. (**j**) Representative images for the *ngr1*^*−/−*^ mice, highlighting the absence of the NgR1 protein expression in clusters of CD19 + B-cells in similar infiltrates of the leptomeninges during the onset of EAE (hatched boxes). (**k**) Representative images for the *ngr1*^*−/−*^ mice, highlighting the strong expression of the NgR3 protein expression in clusters of CD19 + B-cells in similar infiltrates of the leptomeninges during the onset of EAE. Scale = 50 μm.

Following the identification that NgR1 and NgR3 are expressed on a subset of B-cells, we next posited whether receptor expression was dynamic or static in nature and where these B-cells are localized within inflammatory CNS infiltrates. Using western blot analyses of spleen and spinal cord extracts from MOG_35-55_–induced mice at onset of disease (clinical score 1), NgR1 and NgR3 in *ngr1*^+*/*+^*,* and NgR3 in *ngr1*^*−/−*^ mice respectively, were elevated (Fig. 2a). Quantification of NgR1 and NgR3^+^ B-cells per unit area (mm^2^) of spleen and spinal cord inflammatory lesions in *ngr1*^+*/*+^ and *ngr1*^*−/−*^ mice (n = 34 *ngr1*^+*/*+^ mice; 108 sections, n = 15 *ngr1*^*−/−*^ mice; 40 sections) during the onset of EAE was performed to define the number of these cells localized within the respective regions (Fig. 2b).

We showed that the numbers of NgR1^+^ and NgR3^+^ B-cells were significantly increased in infiltrates around the leptomeninges (Fig. 2c-e B220 + and Fig. 2h-I CD19+) at the onset of EAE symptoms induced by the MOG_35-55_ peptide in the *ngr1*^+*/*+^ mice when compared to controls (279.4 ± 43.9 cells per mm^2^ for NgR1 and 336.4 ± 50.1 cells per mm^2^ for NgR3, *p* < 0.001 vs controls for both; Fig. 2b).

We also demonstrated that increased numbers of NgR3^+^ B-cells were also present in the leptomeningeal infiltrates of EAE-induced *ngr1*^*−/−*^ mice while NgR1 was absent (Fig. 2f–g B220 + ; Fig. 2j-k CD19 +), as expected, at the onset of disease (240.2 ± 34.3 cells per mm^2^ for NgR3, *p* < 0.001 vs controls; Fig. 2b). These data demonstrate that NgR1 and NgR3 are expressed on significant numbers of B-cells present within leptomeningeal infiltrates at the clinical onset of EAE and that this expression is inducible.

### BAFF may play a role in stimulating B-cells that express NgR1 and NgR3 during the disease onset of EAE

**Figure 3:**
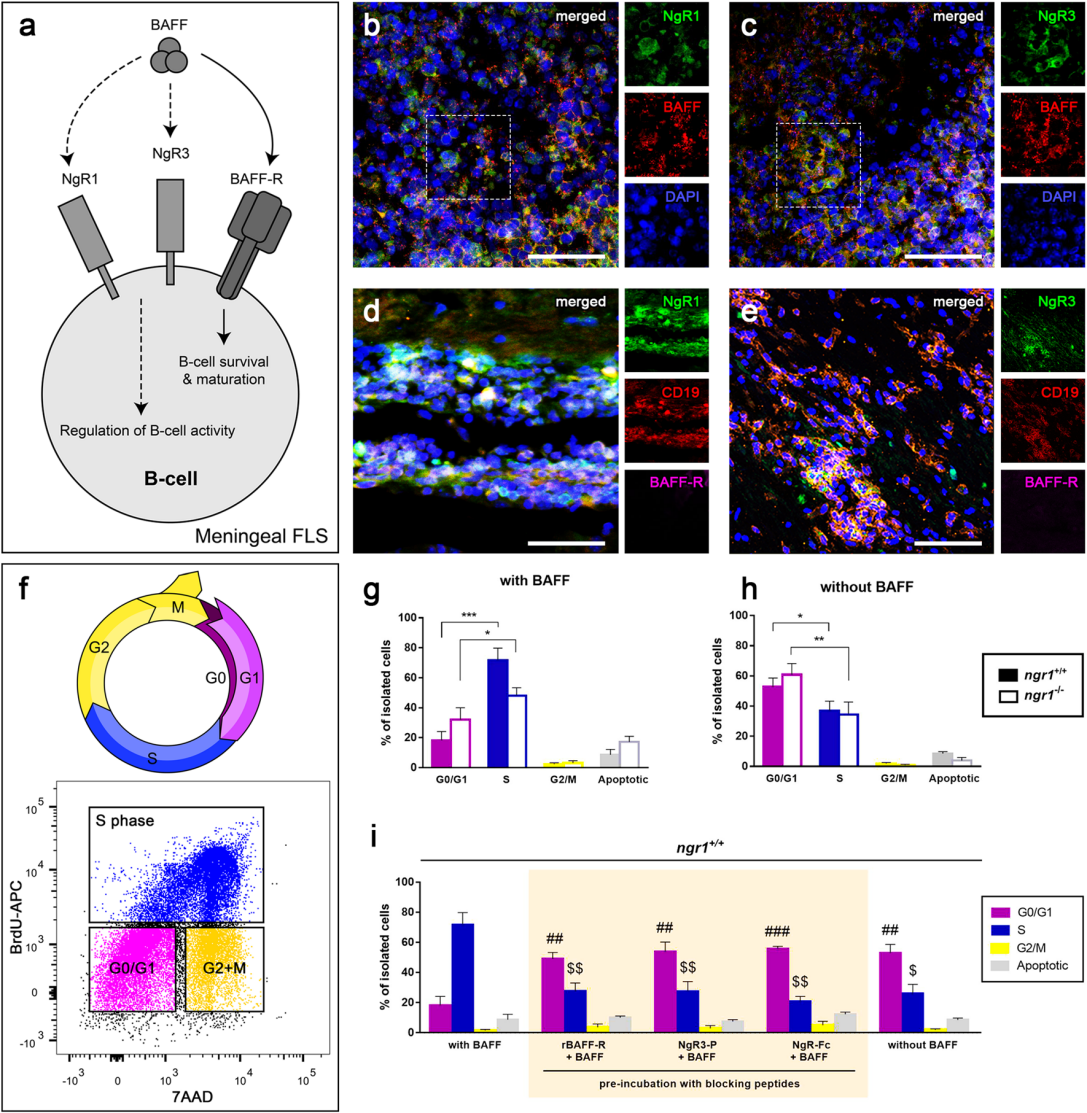
BAFF play a role in stimulating B-cells that express NgR1 and NgR3 during the disease onset of EAE. (**a**) Schematic representation of the potential BAFF/NgR-dependent mechanism operative during the induction of EAE. (**b**,**c**) Substantial numbers of clustered immune cell infiltrates that co-labeled either BAFF and NgR1 or, BAFF and NgR3, were found within the spinal cord of *ngr1*^+*/*+^ EAE-induced mice at disease onset. (**d**,**e**) No double-positive immune cells co-expressing BAFF-R with NgR1 and NgR3 were identified in infiltrates of the spinal cord in *ngr1*^+*/*+^ mice. Hatched boxes are further apposed in separate channels; scale = 50 μm. (**f**) Cell cycle assessment (G1, gap 1 phase; S, synthesis; G2, gap 2 phase; M, mitosis) with flow cytometric analysis showing the gating for the cell cycle phases; G0/G1 (purple color), S phase (Blue), G2/M phase (yellow); BrdU, 5-Bromo-2′-deoxyuridine; 7AAD, 7-aminoactinomycin D (7AAD). (**g**,**h**) NgR1^+^ and NgR3^+^ B-cell population was either stimulated with 50 ng/mL recombinant BAFF (with BAFF) or left untreated (without BAFF). BAFF stimulation led to a significant increase in DNA synthesis (S phase compared to G0/G1 phase; n = 12; *t* test ***p < 0.001) of isolated cells from *ngr1*^+*/*+^ mice while cells remained in the G0/G1 phase (n = 12; *t* test *p < 0.05) in the absence of BAFF. Isolated cells from *ngr1*^*−/ −*^mice also exhibited significant differences between the G0/G1 and S phases in the BAFF-treated cultures (n = 10; *t* test *p < 0.05) and the untreated ones (n = 10; *t* test **p < 0.01), highlighting the role of NgR3 in the absence of NgR1. (**i**) To identify NgR interactions with BAFF, rBAFF was blocked with an excess amount of either rBAFF-R, NgR1-Fc or NgR3-P. All three blocking peptides were efficacious at blocking the cell cycle-dependent BAFF activity when compared to the BAFF-treated cultures. Bars represent mean ± SEM; ^#^*p* < 0.05, ^##^*p* < 0.01, ^###^*p* < 0.001 comparisons with the G0/G1 phase of the BAFF-treated cultures, *t* test (with Bonferroni correction); ^$^*p* < 0.05, ^$$^*p* < 0.01, ^$$$^*p* < 0.001 comparisons with the S phase of the BAFF-treated cultures, *t* test (with Bonferroni correction).

Since BAFF has been previously shown to act as a ligand for Nogo receptor^21^ and we identified the expression of NgR1 and 3 on B-cells within the CNS, an investigation towards a BAFF/NgR-dependent mechanism operative during the induction of MOG_35-55_ EAE, was conducted (Fig. 3a). Spinal cord sections from EAE-induced *ngr1*^+*/*+^ mice were stained for NgR1, NgR3, BAFF and BAFF-R. Substantial numbers of clustered immune cell infiltrates that co-labeled for either, BAFF and NgR1 or, BAFF and NgR3, were identified within the spinal cord of *ngr1*^+*/*+^ EAE-induced mice at disease onset (Fig. 3b,c). However, we could not detect double-positive immune cells that co-expressed BAFF-R with any of the Nogo receptors localized to the leptomeninges of the spinal cord (Fig. 3d,e).

Similarly, BAFF^+^ NgR1^+^ and BAFF^+^ NgR3^+^ immune cell populations were exhibited within the splenic follicles of EAE-induced *ngr1*^+*/*+^ mice at the onset of EAE, while BAFF-R could only be detected on occasional immune populations of the spleen (Additional file 3: Fig. S3a–d, arrows). In order to define how BAFF may modulate B-cells, which express NgR1 and NgR3 during the onset of EAE, cells from the spinal cord and spleen of the *ngr1*^+*/*+^ mice were isolated by FACS, and cultured (spinal cord; n = 12, spleen; n = 10) in the presence or absence of recombinant BAFF (rBAFF) [50 ng/mL]. Stimulation of B-cells positive for either NgR1 or NgR3 with rBAFF led to a significant increase in DNA synthesis (Fig. 3f) [S phase, 71.6 ± 8.2% vs G0/G1 phase 18.2 ± 5.8%, *p* < 0.001; Fig. 3g] compared to the cultures that were not stimulated with BAFF (G0/G1 phase 52.7 ± 5.9% vs S phase, 36.8 ± 6.3%, *p* < 0.05; Fig. 3h). Analogous results were retrieved from *ngr1*^*−/−*^ mice (with BAFF: S phase, 48.1 ± 5.1% vs G0/G1 phase 32.1 ± 7.9%, *p* < 0.05; without BAFF: G0/G1 phase 60.8 ± 7.4% vs S phase, 34.3 ± 8.3%, *p* < 0.01). No significant differences in the percentage of apoptosis were observed with or without BAFF stimulation for both cohorts of mice. Likewise, there were significant differences between the G0/G1 and S phases in the cells isolated from the spleens of *ngr1*^+*/*+^ and *ngr1*^*−/−*^ mice; isolated B-cells were mainly residing in S phase with BAFF stimulation and preferred G0/G1 over S phase when they were devoid of BAFF (Additional file 3: Fig. S3e,f).

To further illustrate this, we identified that isolated cells also remained in G0/G1 phase in the spinal cords of *ngr1*^+*/*+^ mice when BAFF activity was blocked by pre-incubating the culture medium with either (i) rBAFF-R (n = 8 independent experiments) or; (ii) NgR3-peptide (n = 6 independent experiments); or (iii) NgR-Fc fusion protein (n = 6 independent experiments) (Fig. 3i). More specifically, 27.7 ± 5.3% of the isolated cells from the rBAFF-R cohort, 27.5 ± 6.3% from the NgR3-P and only 20.6 ± 3.4% from the NgR-Fc group were identified in the S phase, and were significantly lower in comparison to the control group, treated with BAFF (*p* < 0.01 for all comparisons). Interestingly, NgR3-P and NgR-Fc fusion protein were efficacious at blocking the cell cycle-dependent BAFF activity (53.6 ± 6.5% and 55.8 ± 1.5% of the isolated cells stayed in the G0/G1 phase, respectively) in a similar manner to that achieved with rBAFF-R antagonism (Fig. 3i).

In parallel experiments using cells isolated from spleens, we demonstrated that the NgR3-P and NgR-Fc fusion proteins were extremely efficacious at blocking the cell cycle-dependent BAFF activity halting 86.8 ± 4.7% and 85.5 ± 6.3% of the isolated cells in the G0/G1 phase, while only 7.7 ± 3.6% and 8.0 ± 4.5% could enter the S phase, respectively (Additional file 3: Fig. S3g). These data suggest that both NgR1 and NgR3 are active contributors to B-cell maturation during EAE and in fact may contribute to the signal transduction elicited by BAFF during spinal cord inflammation.

### Immunoglobulin-specific phenotyping of B-cell infiltrates expressing NgR1 and NgR3 during EAE at the onset of EAE

B-cells expressing either NgR1 and/or NgR3 were isolated from the spinal cords of EAE-induced *ngr1*^+*/*+^ and *ngr1*^*−/−*^ mice and phenotyped according to their membrane-bound immunoglobulins, to investigate potential humoral/immunopathogenic roles of NgR.

**Figure 4:**
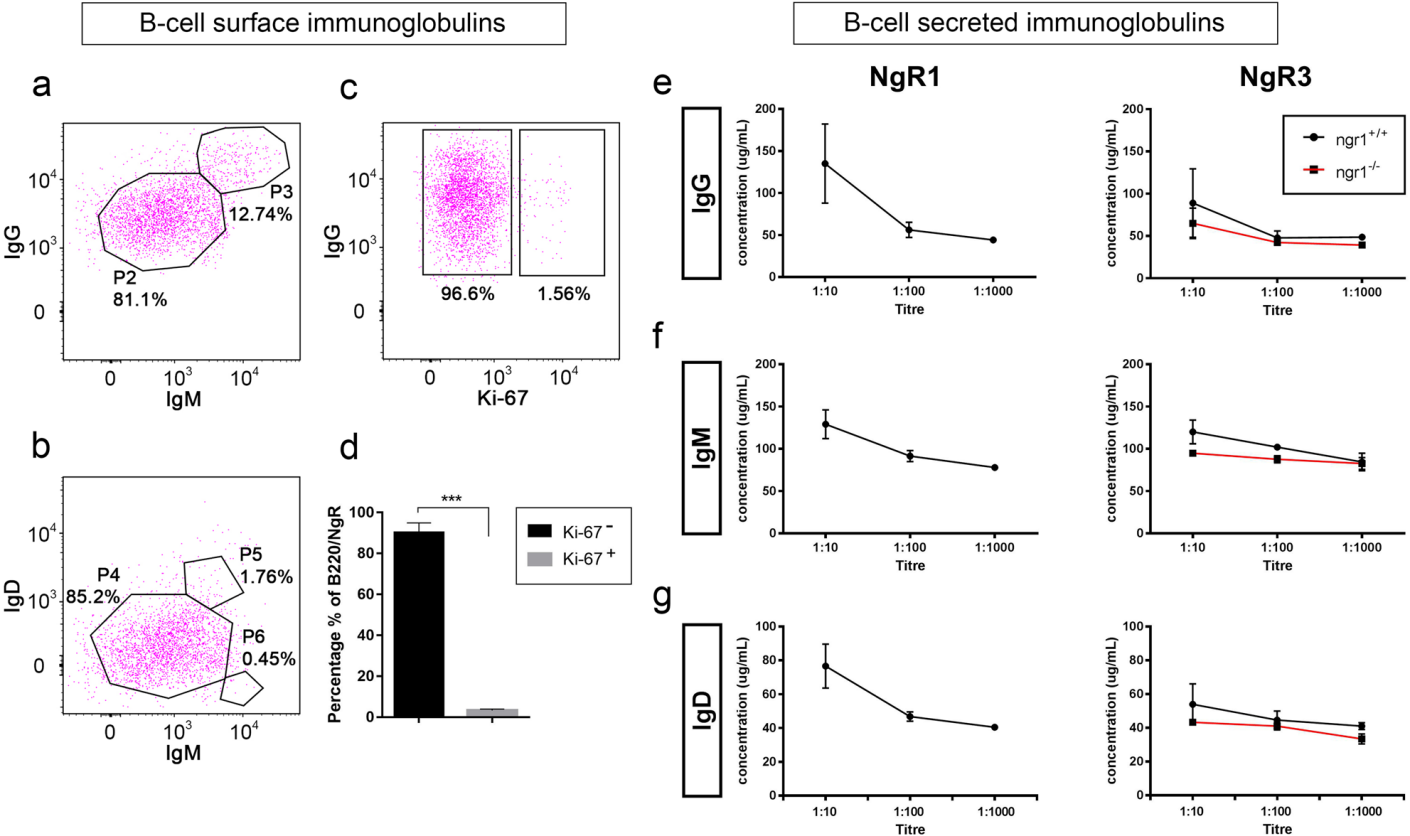
NgR1^+^ and NgR3^+^ B-cells exhibit increased secretion of IgG during the EAE onset. (**a**) Representative dot plots of NgR3^+^ B-cells isolated from both *ngr1*^+*/*+^ EAE-induced mice exhibited increased levels of IgG than IgM on their cell surface. (**b**) IgM^+^ B-cells found to be more abundant in comparison to IgD^+^ B-cell populations. (**c**) NgR1^+^ and NgR3^+^ B-cells were found to be mainly IgG^+^ with the representative estimation of NgR3^+^ isolated B-cells exhibiting ~ 96.6% IgG^+^, when gated against the cell proliferation marker Ki-67. (**d**) The data are represented in a bar graph of multiple experiments with mean ± SEM (n = 6; *t* test ***p < 0.001). (**e**–**g**) The titers of the secreted immunoglobulins from isolated and cultured NgR1^+^ and NgR3^+^ B-cells were also evaluated from isolated supernatant samples by ELISA for both *ngr1*^+*/*+^ and *ngr1*^*−/−*^ mice. Lower titers yielded a higher concentration of the respective antibodies. *ngr1*^*−/−*^ mice showed a reduced capacity for production of IgG, IgM and IgD but with no statistically significant differences. Results represent mean ± SEM.

Flow cytometric analysis was performed on immune cells isolated from lumbar spinal cord at disease onset were stained with fluorescently tagged anti-NgR1 and 3, anti-B220, anti-IgM and IgD antibodies. Representative results showed that the majority of cells (> 80%) expressed high levels of cell surface IgG and low levels of IgM with a minor proportion (~ 12%) co-expressing high amounts of IgG and IgM (Fig. 4a,b). Furthermore, IgD was co-expressed on small proportion (~ 1%) of cells expressing high levels of IgM. Similar results were generated in *ngr1*^*−/−*^ mice. The majority of double-positive IgG^+^ B-cells (96.6%) were not cycling based on Ki-67 labeling (Fig. 4c,d). These data suggest that the isolated spinal cord B-cells from EAE-induced mice that express NgR have matured and underwent immunoglobulin class switching.

Supernatants from the spinal cord and spleen of *ngr1*^+*/*+^ and *ngr1*^*−/−*^ EAE-induced mice at disease onset were collected from sorted and cultured cells before BAFF stimulation, and the IgG, IgM and IgD secreted immunoglobulin profiles were examined using ELISA (Fig. 4e–g). The responses of IgA, IgE and IgG subclasses (IgG1, IgG2b, IgG2c and IgG3) were also evaluated (data not shown).

Upon interrogation of titre levels present within the culture medium supernatants for all the secretory immunoglobulins from the cells isolated from the spinal cord, the dilution that yielded the most abundant fraction was found to be 1:10. Spleen isolates showed comparable efficiency. At this titre, B-cells expressing NgR1 from *ngr1*^+*/*+^ genotype, were found to secrete 135.1 ± 47.1 μg/mL IgG, 129.0 ± 17.0 μg/mL IgM and 76.6 ± 13.0 μg/mL IgD, while B-cells expressing NgR3 from *ngr1*^+*/*+^, were found to secrete 88.9 ± 40.4 μg/mL IgG, 120.0 ± 14.0 μg/mL IgM and 53.9 ± 12.0 μg/mL IgD. In the *ngr1*^*−/−*^ group, we failed to detect any immunoglobulin secreted from NgR1^+^ B-cells but NgR3^+^ B-cells could yield 64.9 ± 18.1 μg/mL IgG, 94.8 ± 2.1 μg/mL IgM and 43.2 ± 0.1 μg/mL IgD although it did not show any significant difference when compared with the titers of the *ngr1*^+*/*+^ group (Mann–Whitney test; *p* > 0.05). Based on these findings, CNS-infiltrating B-cells expressing NgR1 and NgR3 undergo class switching, with a profound IgG humoral response.

### Secreted IgG from ***ngr1***^+^ and NgR3^+^ isolated spinal cord B cells recognize epitopes on myelin and oligodendrocyte glycoproteins

In order to determine whether secreted IgG antibodies generated by NgR1^+^ or NgR3^+^ B-cells isolated from diseased spinal cords were reactive against myelin, immunostaining was performed on spinal cord tissue sections obtained from non-diseased *ngr1*^+*/*+^ mice (naïve controls).

**Figure 5:**
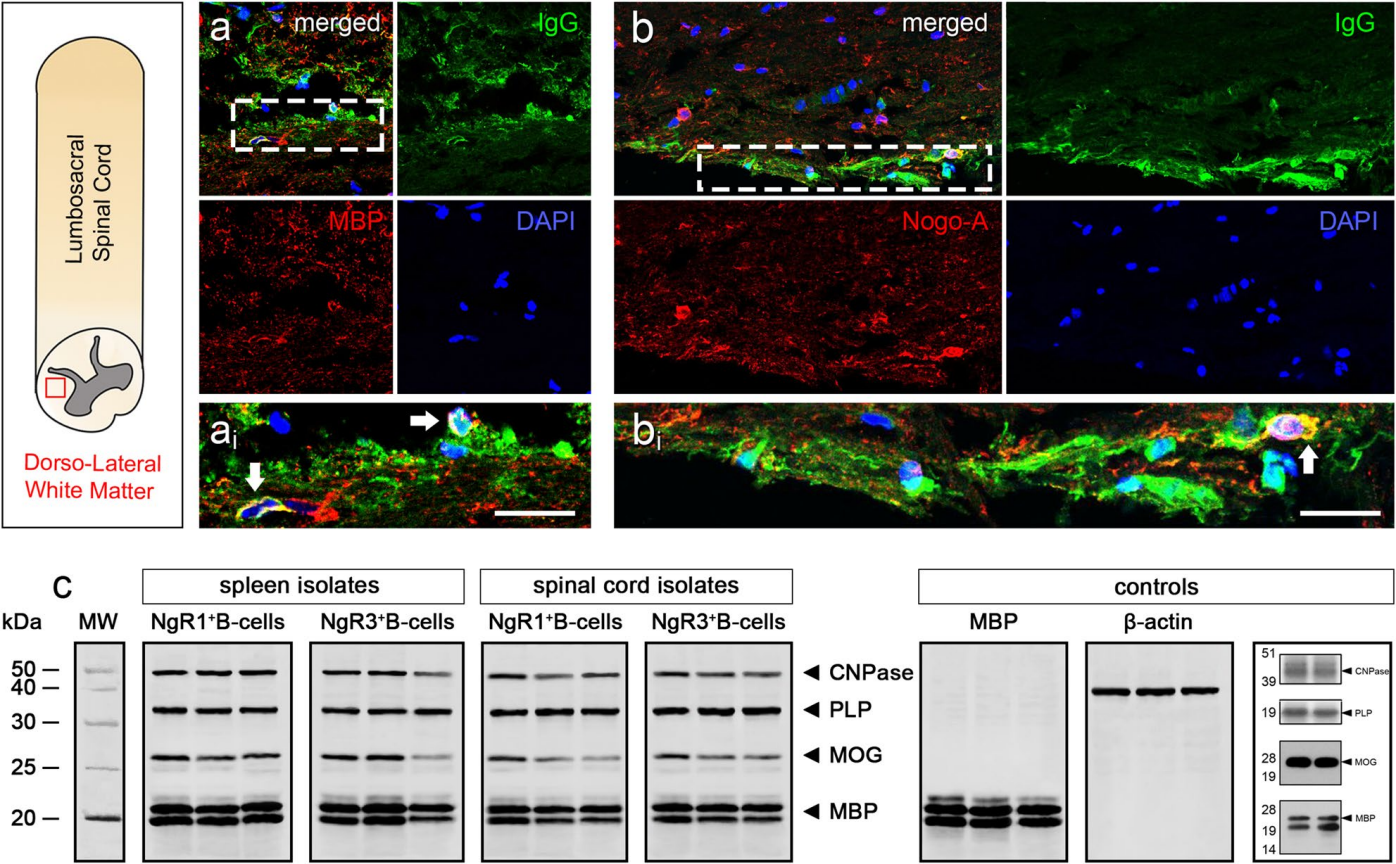
Secreted immunoglobulin responses by BAFF-responsive NgR1^+^ and NgR3^+^ B-cells are directed against CNS myelin. (**a**,**b**) Double immunofluorescence labeling of NgR1^+^ and NgR3^+^ B-cell-secreted IgG (green) co-localized with MBP and Nogo-A, respectively (red), within adjacent spinal cord sections of EAE mice. DAPI (blue) stained cell nuclei. a_i_: inset of hatched box in a, b_i_: inset of hatched box in b. White arrow indicates co-localization. Scale = 5 μm. (**c**) Western Immunoblot analysis of secreted IgG in the supernatant from isolated cells. Purified myelin from lumbar spinal cord of *ngr1*^+*/*+^ and *ngr1*^*−/−*^ mice were differentiated on a 4–12% Bis–Tris gel. IgG presented in the supernatant could directly bind to the major myelin proteins MBP, MOG, PLP and CNPase, annotated in the gel, respectively. The three lanes per box are replicates. No immunoreactive bands were detected when a commercial IgG antibody was utilized as a control.

We identified that clusters of spinal cord white matter MBP^+^ and Nogo-A^+^ cells were colocalized with secreted IgG present within the supernatant from the cultured NgR3^+^ B-cells of EAE-induced *ngr1*^+*/*+^ mice (Fig. 5a,b), suggesting that these antibodies are recognizing antigenic epitopes present on oligodendroglial lineage cells. Interestingly, IgG^+^ and Nogo-A^+^ co-immunoreactivity detected nodal myelin labeling, as demonstrated under high magnification insets (Fig. 5ai,b_i_).

The ability of these secreted IgG antibodies from isolated NgR1^+^ or NgR3^+^ B-cells (which bind to spinal cord white matter myelin), were further examined for their ability to bind to specific myelin epitopes (n = 3 independent experiments). Our results indicated that these IgG anti-myelin antibodies from isolated NgR1^+^ or NgR3^+^ B-cells (from either the spleen or spinal cord of *ngr1*^+*/*+^ mice at clinical score 1) could bind directly to all major myelin glycoproteins including MBP, MOG, PLP and CNPase (Fig. 5c). These results highlight that the secretory form of IgG derived from isolated peripheral and intrathecal NgR1^+^ and NgR3^+^ B-cells, directly interact with myelin, suggestive of a humoral response by BAFF-sensitive NgR1^+^ and NgR3^+^ B-cells.

## Discussion

Current MS treatments predominantly target the inflammatory aspects of the disease, however, chronic-active MS lesions do not respond effectively to monotherapies^6^. This highlights the critical need to develop novel disease modifying agents that can address the gap in treating acute and/or progressive MS with profound neurodegenerative changes. Our previous work has shown that deletion of NgR1 can protect against axonal degeneration, and thus disease progression, in EAE-induced mice^30^. These data were further supported with signal transduction evidence to show that NgR1 may ultimately potentiate neurodegeneration in EAE via a direct mechanism that stalls axonal transport. We identified that despite inflammatory infiltrates present during the peak stage of EAE, conditional deletion of NgR1 in neurons limits axonal degeneration and demyelination^31^. In the current study, we investigated the role of NgR1 and its homologs, specifically during B-cell maturation and differentiation. We identified infiltrates localized to the CNS during the onset of EAE that exhibited NgR1 and NgR3 expression. These NgR1^+^ and NgR3^+^ B-cells were significantly elevated at the acute onset stage of EAE, following immunization of mice either with MOG_35-55_ or rMOG.

For the first time, we identified that both NgR1^+^ and NgR3^+^ are active contributors to B-cell maturation during EAE and may participate in the signal transduction events elicited through BAFF-specific stimulation to enhance the proliferation of B-cells within EAE lesions. Importantly we show that mature secretory NgR1^+^ and NgR3^+^ B-cells can generate immunoglobulins that bind to spinal cord white matter myelin and the integral myelin membrane glycoproteins. Our findings emphasize that NgR1^+^ and NgR3^+^ may contribute to signaling in B-cells during the onset of EAE and can generate autoantibodies against myelin. The relevance of these B-cells in the pathogenesis of inflammatory demyelination that govern MS progression requires investigation since if we could therapeutically target this B-cell population during the course of MS, it may serve as a more specific disease modifying regimen than depleting B-cells on a broader scale.

B-cells play a significant role in MS pathogenesis, in addition to their ability for processing and presenting antigen^46^. Recent clinical trials and archival post-mortem analyses have established the benefit of targeting leptomeningeal-localized B-cell populations to limit MS relapse rates and progression^14,47,48^. However, a recent clinical phase II randomized, double-blind, placebo controlled trial with tabalumab (NCT0088299999), a IgG4 antagonizing monoclonal humanized antibody directed against membrane-bound and soluble BAFF, did not meet its primary endpoint of reduced gadolinium-enhancing MRI lesions in 197 RRMS patients that completed the 73 week study^49,50^. Moreover, the secondary endpoints of T2-weighted lesions and annualized relapse rates were not met. Therefore, the failure of this well powered and designed trial would suggest that antagonizing peripheral BAFF-dependent B-cell activity may not be relevant in limiting immune-mediated demyelination in humans and would advocate for greater mechanistic studies to be performed to understand the signaling dynamics of leptomeningeal follicular B-cells that may propagate inflammatory demyelinating lesions. Since NgR1 and its downstream signaling cascade has been defined as a critical mechanism governing axonal and myelin degeneration during the onset and progression of EAE, we set out to address the main question of how the receptor may influence immunopathogenic mechanisms (if any) that govern this model of inflammatory demyelination.

We have shown that specific B-cell populations express NgR1 and NgR3 clustered within leptomeningeal infiltrates of the lumbosacral spinal cords during the onset of EAE-induced inflammation. This finding was supported by data showing that antagonizing BAFF-dependent B-cell stimulation can be achieved by the provision of the NgR-Fc and NgR3-P fusion proteins, blocking B-cell proliferation. A previous study has demonstrated that NgR1 and NgR3, co-expressed on 293 T cells, can collaborate in the same receptor complex^51^. In CNS tissue, neurons have been shown to upregulate the mRNA expression of NgR3 during kainic acid treatment^52^, with chondroitin sulphate proteoglycans (CSPGs) able to ligate to NgR3 to regulate neurite outgrowth inhibition^51^. Importantly, it has been demonstrated that ablation of the putative CSPG receptor type protein tyrosine phosphatase (Rptpσ) can enhance axonal regeneration following optic nerve crush injury^51^. Although the role of NgR3 has not been defined in B-cells, Rptp (CD148) signaling has been demonstrated to regulate B-cell and macrophage lineage cell differentiation^53^. Moreover, CSPGs derived from either T-cells or reactive astrocytes are capable of stimulating B-cell differentiation^54^ and may play a role for antigen presentation during autoimmunity^55^ or even capable of binding APRIL to regulate B-cell maturation^22^. These data raise the tantalizing hypothesis that NgR1 and NgR3 may collaborate with various receptors in multimeric complexes regulating signal transduction cascades for B-cell activation. This is of particular importance in MS since the histopathological observations have demonstrated that B-cells are indeed presented in follicles at the meninges of MS patients^47,56^ and may regulate progressive neurodegeneration^57^.

The question whether or not NgR expression in B-cells and downstream signaling govern the formation of follicular-like structures in the CNS during progressive MS requires elucidation since our current findings implicate NgR1 and NgR3 as collaborative players in the induction of EAE. Although the exact mechanism by which B-cells contribute to the cellular and humoral responses that govern MS pathogenesis is still not fully understood, B-cell-targeted therapies have shown promising outcomes in clinical trials^3,58^ and so if the expression of NgR1 and NgR3 can be therapeutically targeted, then novel pathways for modifying disease specific B-cells may have been identified considering our current data. However, recent novel data generated in the EAE model of MS show that IgA generated from plasma cells localized to the CNS are derived from the gut^58^. This study has intriguingly demonstrated that removal of this population of IgA + plasma cells exacerbated EAE implicating their neuroprotective nature driven by IL10. Specifically, this study demonstrated that BAFF transgenic mice that overexpress BAFF and exhibit increased levels of circulating IgA, were resistant to the induction of MOG_35-55_ and recombinant human EAE^59^. These authors concluded that the Transgenic overexpressing BAFF studies along with the observations from TACI knock out mice (*Tnfrsf13b-/-*), may suggest that BAFF signaling through TACI provides a protective effect to limit immune-mediated demyelination through the maintenance of IgA + plasma cells in the gut and the CNS. Our data would support the notion that at peak stage of EAE we did not demonstrate high levels of IgA but only IgM and IgG, that we demonstrated could be generated from NgR1 + and NgR3 + B cells grown in vivo. Whether these Nogo Receptor expressing B cells can eventually derive BAFF-responsive IgA + Plasma cells remains to be determined.

With the emerging view that BAFF is a known stimulator of B-cell activation^18–20^, our investigations identifying the BAFF responsiveness of NgR1^+^ and NgR3^+^ B-cells during EAE induction, raises key questions surrounding the pathophysiological role of these receptors. Of particular importance is that BAFF has been highlighted as a potential target in MS because it is produced endogenously in the CNS by astrocytes during the pathogenesis and it is also associated with BAFF-R expressing cells, which are up-regulated within the meninges in ectopic lymphoid follicles^16,60^. Whether NgR1 and NgR3 can amplify the signaling of BAFF in B-cells expressing these receptors is yet to be established. However, our data has identified that we can block the B-cell proliferative response by antagonizing BAFF with recombinant NgR peptides implicating a signaling role.

It has been identified that BAFF can act as an alternate ligand for NgR1 to inhibit DRG neurite outgrowth in vitro^21^. However, our study is the first to demonstrate that there exists a BAFF-dependent B-cell pathophysiological response that is driven through NgR1 and NgR3. Our finding that BAFF can stimulate spinal cord-specific NgR1^+^ and NgR3^+^ B-cells to proliferate, confirm a signaling role for these receptors during neuroinflammation. Importantly, without BAFF stimulation these NgR1^+^ and NgR3^+^ B-cells were also able to generate IgG antibodies either expressed on the surface (defining maturation through class switching), or secreted as IgG myelin-specific antibodies. Whether these autoantibodies are capable of promulgating neurodegeneration or targeted deletion of NgR1^+^ and NgR3^+^ B-cells during neuroinflammation can ameliorate disease progression requires further investigation.

The current study has identified for the first time that both NgR1 and NgR3 are active contributors to B-cell maturation during EAE and may participate in the signal transduction events elicited through BAFF ligation that may enhance proliferation of these cells intrathecally. Although the direct signal transduction events within these B-cells are yet to be elucidated, the responsiveness of the NgR1^+^ and NgR3^+^ B-cells may be a major pathogenic event governing the cycle of neuroinflammation. The pathobiological consequences of myelin-specific immunoglobulins generated by this population of B-cells may be of great significance during the epitope spreading effects of EAE-induced neurodegeneration and by extension MS, raising the possibility of novel B-cell therapeutics targeting NgR1 and NgR3.

## Materials and methods

### Mouse breeding

All experiments were implemented in accordance with the guidelines of the Australian code of practice for the use and care of animals for scientific purposes and approved by the Monash University Animal Ethic Committee (Alfred Medical Research and Education Precinct; AMREP) and office of the Gene Technology Regulator of Australia (AEC AMREP Approval number E/1532/2015/M). Female *ngr1*^*−/−*^ mice (aged 8–12 weeks) and their *ngr1*^+*/*+^ littermates were bred and maintained at the AMREP animal services. Exon 2 of the *ngr1* gene was a double-targeted allele on a C57Bl/6 background and backcrossed more than 10 generations as mentioned previously^61^.

### EAE induction and clinical assessment

In accordance with the ARRIVE 2.0 guidelines, this original study compared EAE clinical scores, immune cell profiles and histopathological outcomes in 8–12 weeks old C57Bl/6 (*ngr1*^+*/*+^ or *ngr1*^*−/−*^) female mice with or without MOG_35-55_-induction using a blinded and randomised study design. To induce EAE, 8–12 weeks old C57Bl/6 (*ngr1*^+*/*+^ or *ngr1*^*−/−*^) female mice were immunized with either 200 μg MOG_35-55_ (Purar Chemical, China) or full-length recombinant mouse MOG (AnaSpec, USA) as previously described^30,31,62^. Mice with complete Freund’s adjuvant but without MOG peptide (CFA only), served as non-specific immunization controls. All EAE animals were examined for 28 consecutive days using a six-grade score scale consistent with well documented disease stages as previously described^63,64^: 0, asymptomatic; 1, partial loss of tail tonicity; 2, flaccid tail paralysis; 3, difficulty to roll over from a supine position; 4, hindlimb paralysis; 5, forelimb paresis. Animals without a score were excluded from further neuropathological analysis. The disease stages classically included: pre-onset (0–10 days post immunization (dpi); score 0), onset (10–20 dpi; mean score 1), peak (20–30 dpi; mean score 3). The following indexes were also calculated: Mean day of disease onset (dDO), mean maximal score (MMS) and area under the curve (AUC), which cumulatively measure the extent and burden of the disease. All groups of *ngr1*^+*/*+^ and *ngr1*^*−/−*^ mice experimental groups were coded to perform a blinded study, to limit observer bias when assessing EAE clinical score. The codes and data were stored electronically and as hard copies by the senior investigator (SP) and then decoded upon collection of all data synthesized for analysis.

### Tissue processing

Mice were euthanized at various stages of EAE via CO_2_ inhalation according to ethical guidelines. Spleens and spinal cords were dissected to isolate immune cells for immune-phenotyping analysis. Otherwise, an equal amount of 4% paraformaldehyde (PFA) fixative was injected into the left ventricle and tissues were then processed for immunohistochemistry. Longitudinal spleen and spinal cord sections were cut into 10 μm serial sections and processed for immunohistochemistry.

### Isolation of immune cells and myelin from mouse tissues

#### Spleen isolation

B- and T-cells were isolated from the spleens of EAE-induced and non-immunized *ngr1*^+*/*+^ and *ngr1*^*−/−*^ mice. Tissues were mashed by passing through a 40 μm nylon filter, red blood cells were lysed with red blood cell lysis buffer (Sigma-Aldrich) and the remaining cells were washed in D-PBS and subjected to flow cytometry analysis.

#### Spinal cord isolation

Spinal cord tissue was chopped into fine pieces and then enzymatically digested at 37 °C for 30 min with collagenase D (Roche)/Dnase I (Roche) in PBS. These were then subjected to Ficoll density gradient (Stem Cell Technologies). The gradient interface was collected upon centrifugation at 800 g max for 30 min at 20 °C. The remaining cells were analysed by flow cytometry analysis.

#### Myelin isolation

Purification of central nervous system myelin was performed as described previously^65^. Lumbosacral spinal cord was obtained from both *ngr1*^+*/*+^ and *ngr1*^*−/−*^*,* immunized with MOG_35-55_ and non-immunized mice. Fresh frozen spinal cord (immersed in liquid N_2_) was then grounded to a fine powder in liquid N_2_ and homogenized in 0.29 mol/L of sucrose solution (5% w/v). After preparing a discontinuous sucrose gradient by layering aliquots of the homogenate over of 0.85 mol/L sucrose, this was then spun for 45 min at 90,000 g max. The interface, including crude whole myelin, was collected and diluted 1:1 with 0.24 mol/L sucrose. Then this was spun for 30 min at 17,000 g max to collect the pellet, which was resuspended in dH_2_O (osmotic shock), and re-spun again for 30 min at 17,000 g max. A second discontinuous sucrose gradient was performed for further myelin purification, and to remove any immunoglobulin contamination that may provide a false positive signal. The purified myelin was treated with diethylamine-HCl (50 mmol/L, pH = 11.5), then pelleted in a microfuge, and thoroughly washed with dH_2_O to become lyophilized.

### Flow cytometry analysis

Isolated cells were analyzed using flow cytometry to determine whether there existed disparate populations within disease-originating organ sites. Briefly, cells were washed with 1 mL FACS wash buffer [0.02% sodium azide, 1% bovine serum albumin (BSA) in PBS], centrifuged (3x) at 1500 rpm for 5 min. Mouse immune cell antigens were detected using the following fluorescently labeled antibodies (for a full list of antibodies used in all experiments see Additional File 4: Table S1: CD45/B220-PE (BD Biosciences) and its isotype control (BD Biosciences; PE labeled rat IgG 2b kappa); CD3e-Pe-Cy7 (eBioscience); CD11c-APC (BD Bioscience) and its isotype control (eBioscience; PE-Cy7 labeled hamster IgG); NgR1 (R&D systems) and its isotype control (BD Pharmingen; rat IgG 2b kappa); NgR2 (Santa Cruz Biotechnology); NgR3 (Santa Cruz Biotechnology); IgM-PerCP/Cy5.5 (BioLegend); IgD-APC (BioLegend), IgG-Biotin (eBioscience); Streptavidin APC (eBioscience): and Ki-67-eFluor 450 (eBioscience).

In addition to the single staining, each immune cell antigen was stained with the NgR homologs, in order to determine its co-expression on these isolated immune cells. Briefly, normal rat serum (Life Technologies) was added into all tubes to block non-specific binding and incubated at 4 °C for 30 min. The cells were then washed (3x) and centrifuged at 1500 rpm for 5 min at 4 °C, followed by the secondary antibody staining (Alexa fluor 488 goat anti-rat IgG (H + L); Life Technologies) at 4 °C for 30 min. Stained cells were washed and DAPI was added into each sample to label dead cells for 10 min before analysis. Finally, all tubes were analyzed by using the BD FACS Canto II (BD Biosciences).

### Immunohistochemistry

#### Paraffin sections

Fixed (4% paraformaldehyde – PFA), paraffin-embedded tissues were cut into 10 μm serial sections on a conventional microtome and processed for immunohistochemistry. The sections were de-waxed and incubated with proteinase K (20 μg/mL) (Qiagen) for 1 h at 37 °C, then washed (3 × 10 min) with PBS. All sections were post-fixed with 4% PFA for 30 min at room temperature, followed by three washes with PBS. Tissues were then incubated with blocking buffer (5% goat serum, 0.1% triton X-100 in PBS) at 4 °C overnight. All sections were incubated with primary antibodies: anti-BAFFR – Biotinylated (R&D Systems); anti-CD19 (Alexa Fluor 594) (BioLegend); anti-CD45/B220 (BIOSS); anti-BLyS/BAFF (Novus Biologicals); anti-CD268/BAFF Receptor (eBioscience); anti-NgR1 (R&D systems and/or Millipore); anti-NgR2 (Santa Cruz Biotechnology); and anti-NgR3 (Santa Cruz Biotechnology) for overnight at 4 °C, followed by washing (3x) in PBS for 10 min. Appropriate secondary antibodies (Alexa Fluor 488 goat anti-rabbit IgG (H + L) antibody; Life technologies, Alexa Fluor 488 goat anti-rat IgG (H + L) antibody; Life technologies, Alexa Fluor 555 goat anti-rabbit IgG (H + L) antibody; Life technologies, Alexa Fluor 555 goat anti-rat IgG (H + L) antibody; Life technologies, Donkey anti-Goat 488 antibody; Life technologies, Donkey anti-Goat 555 antibody; Life technologies) were applied, and incubated for 2 h at room temperature. Streptavidin (BD Pharmingen) was added to the appropriate slides for 1 h at RT. Finally, DAPI was added after washing in PBS for 15 min and slides were cover slipped using fluorescent mounting medium (Dako). Subsequently, all tissue specimens were captured by the four-channel fluorescence imaging, using a confocal Nikon C1 upright microscope with 40 × and 60 × UPlanApo objective lens.

#### Frozen sections

OCT-embedded tissue blocks were cut into 10 μm serial sections on a cryostat (Leica Microsystems). The sections were post-fixed with 4% PFA for 30 min at room temperature, followed by three washes with PBS. Tissues were then incubated in the blocking buffer for 1 h at room temperature. All sections were incubated with primary and secondary antibodies, followed by DAPI, as indicated above. These were then scanned under an inverted confocal microscope (described above).

### Quantification of NgRs and B-cell populations in the white matter tracts of the lumbar and sacral spinal cord

Analysis of cell numbers localized within specific tissue areas were performed on all spinal cord sections of both *ngr1*^+*/*+^and *ngr1*^*−/−*^ mice following immunohistochemistry. The number of double stained cells (NgR that is expressed on B-cells) was counted per unit area (per μm^2^) of spinal cord longitudinal-sections in all genotypes (3 different sections per mouse > 50 μm apart). The mean and standard error of the mean (SEM) was calculated for each group of mice at different clinical stages of the disease.

### Demyelination and axonal degeneration

Post-tissue processing, Luxol fast blue/Periodic acid-Schiff (LFB/PAS) and Bielschowsky staining were used on spinal cord sections of *ngr1*^+*/*+^ and *ngr1*^*−/−*^ mice at different clinical scores (Histology platform, Monash University) in order to demonstrate areas of demyelination and axonal injury in EAE-induced mice, respectively.

LFB/PAS was performed by dehydrating the tissue sections using 95% ethanol, then placed in Low Viscosity Nitrocellulose (LVN) for 1 min. The sections were then placed into 0.1% LFB solution (w/v) overnight at 40 °C incubator (cover staining dish in cling wrap to avoid excessive evaporation of the solution). Excess stain was rinsed off in 70% ethanol then washed in dH_2_O. The sections were briefly dipped in dilute lithium carbonate (0.5 mg/mL) then differentiated in 50–70% ethanol. Immediately, all sections were washed in dH_2_O (to stop the differentiation but if it over differentiated, were placed back into LFB solution for 5–6 h or overnight). The sections were counterstained with PAS for 5 s, then dehydrated rapidly through ethanol into xylene, then mounted in DPX.

Bielschowsky staining were performed by adding sections into 20% silver nitrate (w/v), incubated for 20 min within a 40 °C incubator. All sections were then washed with dH_2_O and returned into silver nitrate again. Ammonium hydroxide was added drop-by-drop until the precipitate formed had completely dissolved. Then drops of developer stock solution (37–40% formaldehyde, 0.5 g of citric acid (5 mg/mL), 2 drops of 20% nitric acid, and 100 mL of dH_2_O) were added to 20% of ammoniacal silver, then reduced to a visible metallic silver. The sections were dehydrated rapidly through ethanol and then xylene.

### Protein biochemistry

#### Preparation of cell and tissue lysate for western blotting

Spleen and spinal cord samples were ground and lysed from both naïve *ngr1*^+*/*+^ and *ngr1*^*−/−*^ mice using radioimmunoprecipitation assay (RIPA) buffer (Cell Signaling Technology), including PhosSTOP phosphatase inhibitors cocktail (Roche Applied Science) and 1% protease inhibitor (v/v) (Sigma-Aldrich) in a dounce homogenizer. The protein supernatants were collected from homogenates, after centrifuging them at 13,000 g max for 15 min at 4 °C. Protein concentrations were determined by using the bicinchoninic acid (BCA) protein assay kit (Thermo Fisher Scientific).

### Western immunoblotting

10 μg of protein isolated from spleen, spinal cord and myelin samples were loaded into 4–12% graded sodium dodecyl sulphate polyacrylamide gels (SDS-PAGE) (Invitrogen). The proteins were blocked with 5% skim milk powder (w/v) in Tris-buffered saline-Tween (TBST) for 30 min at room temperature after transferring onto polyvinylidene fluoride (PVDF) membranes. The membranes were then incubated with primary anti-NgR1 antibody (Millipore and/or R&D systems), anti-NgR3 antibody (Santa Cruz Biotechnology), anti-Nogo-A (Merck-Millipore) and anti-myelin basic protein (MBP) antibody (Sapphire Bioscience) in 5% skim milk (50 mg/mL) at 4 °C, overnight. The membranes were then washed (3x) with 0.1% TBST for 10 min, followed by adding the secondary anti-rabbit antibody (HRP-conjugated; Merck-Millipore), anti-rat antibody (HRP-conjugated; Merck-Millipore), and anti-mouse antibody (HRP-conjugated; Merck-Millipore) diluted in 5% skim milk or TBST and incubated for 2 h at room temperature; washed (3x) in 0.1% TBST (v/v). The membranes were then incubated with 2 mL of Luminata Forte Western Reagent (Millipore) or Luminata ECL chemiluminescence (Merck-Millipore) for 3–5 min at room temperature for development. Using the Alpha Imager (Alpha Innotech, San Leandro), the films were scanned, and the intensities of the bands were determined using ImageQuant TL v2003 software (Nonlinear Dynamics Ltd, All Saints, Newcastle, UK). Some of the membranes were stained with coomassie blue (8 mg/mL) dye for 30 min to 1 h, and they were scanned after de-staining.

### Cell culture

Immune cells were isolated from spleen and spinal cords of EAE mice, as previously described^30^. The cells were suspended into 2 mL FACS buffer (2% heat inactivated fetal calf serum (FCS) and DPBS) in 5 mL polypropylene round bottom tubes, then stained using only anti-CD45/B220-PE antibodies and either anti-NgR1 or anti-NgR3 antibodies. The double-positive cells were stained with DAPI, then sorted using either BD FACS ARIA or BD Influx sorter. The sorted cells were collected and cultured for 2–3 days in RPMI medium supplemented with 10% heat inactivated FCS (Clontech), 50 μM β-mercaptoethanol, L-glutamine (2 mM), 100 U/ml penicillin, and 100 U/ml streptomycin (Gibco) at 37 °C with 5% CO_2_ in a humidified incubator.

### Cell cycle analysis

In order to determine the ability of BAFF to stimulate B-cells expressing NgR1 or NgR3 in the spleen and spinal cord of *ngr1*^+*/*+^ mice (n = 12) at disease onset, these isolated cells were analyzed with or without BAFF (50 ng/mL) in vitro. Briefly, 50 ng/mL of soluble recombinant mouse BAFF (rBAFF; Enzo) was added into all B-cell cultures and incubated for 24 h at 37 °C in a 5% CO_2_ humidified incubator. To identify NgR interactions with BAFF, rBAFF was blocked with an excess amount of either rBAFF-R, NgR1-Fc or NgR3-P by incubating them for 30 min at 37 °C. The supernatant was collected after spinning for 5 min with 16,000 g max and added to cultured cells for 24 h. 100 μL of supernatant was collected for all samples before and after stimulation with rBAFF, or following inhibition of rBAFF activity with either NgR1-Fc or NgR3-P peptides. The following day, the cell cycle was tested using a BrdU APC flow kit (BD Bioscience). First, 1 mM solution of BrdU was added for 1 h to cultured and treated cells. Second, BrdU-pulsed cells were transferred into FACS tubes and their counts were measured. Antibodies (CD45/B220, NgR and NgR3) were used to stain cells, described above. Cells were fixed and permeabilized with a BD Cytofix/Cytoperm Buffer for 20 min on ice, then washed with 1X BD Perm/Wash Buffer and spun for 5 min at 300 g max. Then the supernatant was discarded. Cells were next incubated with BD Cytoperm permeabilization buffer Plus for 10 min on ice and washed with 1X BD Perm/Wash Buffer again. Cells were re-fixed with BD Cytofix/Cytoperm buffer for 5 min on ice, then washed. Dnase was added into treated cells and incubated for 1 h at 37 °C, then washed with 1X BD Perm/Wash buffer. Finally, cells were stained using an anti-BrdU antibody for 20 min. These were stained with total DNA for cell cycle analysis, 7-aminoactinomycin D (7AAD), after washing it with 1X BD Perm/Wash buffer. Stained cell counts were acquired from the BD LSR II flow cytometer (BD Biosciences).

### Enzyme-linked immunosorbent assay (ELISA)

Supernatant was collected from FAC sorted and cultured cells of *ngr1*^+*/*+^ and *ngr1*^*−/−*^ mice (at clinical score 1 and 3) and then the immunoglobulin class was measured by Enzyme-linked immunosorbent assay (ELISA) with the supernatant diluted as follows: 1:10, 1:100 and 1:1000 for anti-IgG, -IgM and -IgD AP conjugated secondary antibodies (Southern Biotech). The 384-well assay plate (Corning) was coated with 20 µL/well of 2 µg/mL coating reagent (goat anti-mouse Ig (H + L)) (Southern Biotech) and incubated overnight at 4 °C. The plate was washed (2x) with wash buffer (0.1% Tween-20 (w/v), PBS) and (1x) with PBS. 100 µL/well of Block buffer (4% BSA (w/v), PBS) was added and incubated 30 min at room temperature, followed by two washes with wash buffer and one wash with PBS. 40 µL of diluted samples (supernatant collected from sorted and cultured cells) or control antibodies in a dilution buffer (0.5% (w/v) BSA, PBS) were added by serial dilution into the plate into serial dilution (1:10, 1:100 and 1:1000) for the supernatant samples and (1:2) for all control antibodies (Southern Biotech). At the same time, 20 µL of a dilution buffer was added to other wells. Then the plate was incubated for 30 min at room temperature on the plate shaker, followed by two washes with wash buffer and one wash with PBS. 20 µL/well of secondary antibody (diluted 1:2000 in a dilution buffer) (Southern Biotech) was added and incubated for 30 min at room temperature on the plate shaker. Then the plate was washed (4x) with wash buffer and (2x) with PBS. 20 µL/well of 1 mg/mL substrate solution (PNPP tablets dissolved in M.H_2_O; one 20 mg tablet per 20 mL M.H_2_O) (Sigma). Once colorimetric development was achieved, the absorbance measurement was performed at 405 nm using a microtitre plate reader (Bio-Rad Laboratories, CA, USA).

### Statistical analysis

Statistical analysis was carried out using GraphPad Prism 8 software. Values were expressed as mean ± standard error of the mean (SEM). Normality was assessed by D’Agostino–Pearson test. Student’s *t* test and One-way ANOVA followed by Bonferroni *post-hoc* correction, were used for comparisons of 2 or more groups, respectively. In case of non-parametric data, Mann–Whitney or the Kruskall–Wallis test followed by Dunn’s multiple comparison were appropriately applied. Two-way repeated measures ANOVA was used to compare EAE progression in *ngr1*^+*/*+^ and *ngr1*^*−/−*^ mice. *p* < 0.05 was the cut-off level of significance.

## Supplementary Information

Supplementary Figures.

Supplementary Table S1.
