# Supplementary material for: B-cells expressing NgR1 and NgR3 are localized to EAE-induced inflammatory infiltrates and are stimulated by BAFF
Source: Sci Rep. 2021 Feb 3;11:2890. doi: 10.1038/s41598-021-82346-6 (PMC7858582; doi:10.1038/s41598-021-82346-6)
Supplement: Supplementary file 1 — Supplementary Figures. [file 41598_2021_82346_MOESM1_ESM.pdf]

SUPPLEMENTARY INFORMATION

B-cells expressing NgR1 and NgR3 are localized to EAE-induced inflammatory infiltrates and are stimulated by BAFF

Maha M Bakhuraysah[1,2#], Paschalis Theotokis[3#], Jae Young Lee[1,4], Amani A Alrehaili[1,2], Pei-Mun Aui[1], William A Figgett[5], Michael F Azari[1], John-Paul Abou-Afech[1], Fabienne Mackay[5], Christopher Siatskas[6], Frank Alderuccio[7], Stephen M Strittmatter[8], Nikolaos Grigoriadis[3], Steven Petratos[1*]

[1]Department of Neuroscience, Central Clinical School, Monash University, Prahran, Victoria 3004, Australia

[2]Faculty of Applied Medical Sciences, Taif University, Taif 26521, Kingdom of Saudi Arabia

[3]B' Department of Neurology, Laboratory of Experimental Neurology and Neuroimmunology, AHEPA University Hospital, Thessaloniki 54636, Macedonia, Greece

[4]Toolgen Inc., Gasan Digital-Ro, Geumcheon 08594, Seoul Korea

[5]The Peter Doherty Institute, Department of Microbiology and Immunology, School of Biomedical Science, University of Melbourne, Victoria 3000, Australia

[6]STEMCELL Technologies, Vancouver BC, V6A 1B6 Canada

[7]Department of Immunology and Pathology, Central Clinical School, Monash University, Prahran, Victoria 3004, Australia

[8]Program in Cellular Neuroscience, Neurodegeneration and Repair, Yale University School of Medicine, New Haven, CT 06536, USA

#These authors contributed equally to this manuscript

*Correspondence to: Dr Steven Petratos

e-mail: steven.petratos@monash.edu

tel: +613 9902 0191

ORCID of Corresponding author: 0000-0003-1211-4577

ORCID of First author: 0000-0001-8607-6695

Supplementary information

Additional file 1: Figure S1. shows the clinical course of rMOG-induced EAE, the histopathology and the flow cytometric analysis of double-labeled T- and B-cells for NgR1, NgR2 and NgR3, isolated from the spleen and the spinal cord of *ngr1*$^{+/+}$ *and ngr1*$^{-/-}$ mice, respectively. **Additional file 2: Figure S2.** shows the representative plots and histograms of double-labeled T- and B-cells for NgR1, NgR2 and NgR3, form the spleens and spinal cords of EAE-induced *ngr1*$^{+/+}$ *and ngr1*$^{-/-}$ mice, respectively. **Additional file 3: Figure S3.** shows the colocalization of either BAFF or BAFF-R with the NgR homologues in spleen extracts and the results of functional experiments such as the cell cycle analysis with the provision of BAFF and after pre-incubating with blocking peptides.

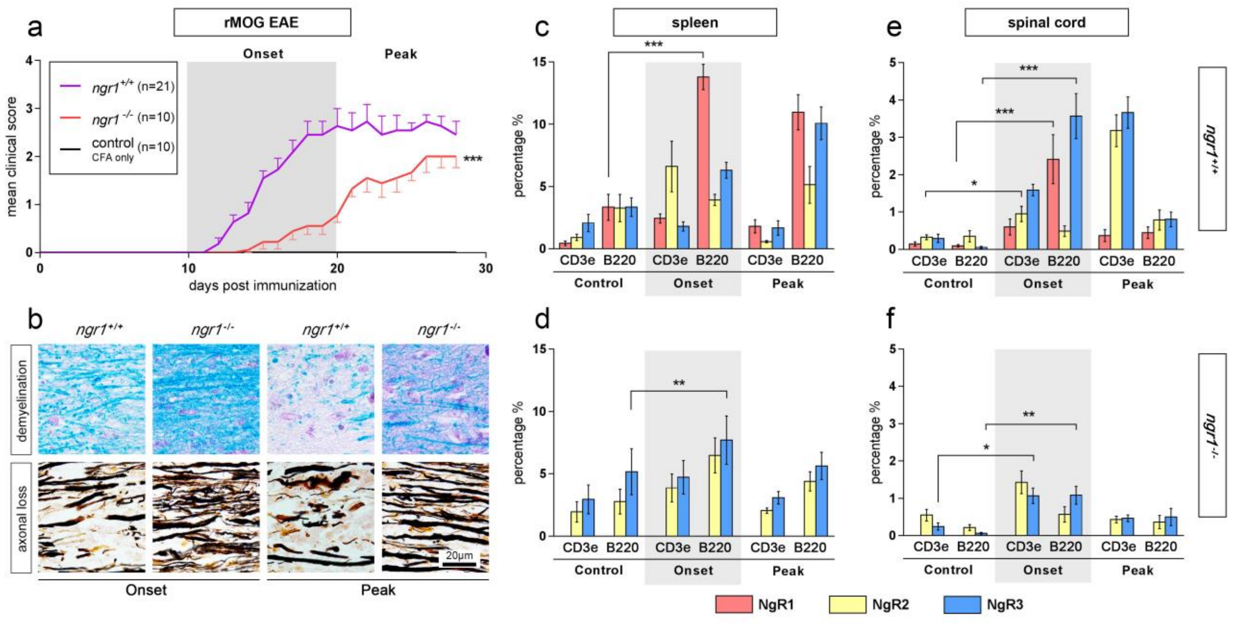

Additional file 1: Figure S1 The onset of rMOG-induced EAE mice is accompanied by an increase in NgR1^{+} and NgR3^{+} immune cell populations. **a** There was a significant delay in EAE onset and reduction in severity for the *ngr1*$^{-/-}$ mice (n=10; red line) compared to *ngr1*$^{+/+}$ mice (n=21; purple line) within the 2 main phases studied; onset (days 10-20 post-immunization; 10-20 dpi) and peak (days 20-30 post-immunization; 20-30 dpi). A control group (CFA-injected only; n=10; black line) was also included. Daily clinical scores represent mean ± SEM, ***p<0.001 (from day 15 until day 25), two-way ANOVA. **b** Reduction in demyelination and axonal loss was observed in the spinal cord of *ngr1*$^{-/-}$ EAE mice, for both onset and peak phases of clinical disease, determined by Luxol fast blue (LFB)/Periodic acid-Schiff (PAS) and Bielschowsky silver stain, respectively. **c, d** Flow cytometric analysis of double-labeled cell suspension from the spleen of *ngr1*$^{+/+}$ mice showed elevated NgR1^{+} B-cells at the onset of EAE (n=5; *t* test ***p<0.001 compared to controls) and a concomitant increase of NgR3^{+} B-cells in *ngr1*$^{-/-}$ (n=5; *t* test **p<0.01). The NgR2 homolog did not exhibit significant modulation in either *ngr1*$^{+/+}$ or *ngr1*$^{-/-}$ mice. **e, f** Spinal cord percentages of double positive B-cells were again significantly higher for NgR1 and NgR3 during the onset of EAE, compared to controls (n=5; *t* test ***p<0.001 for both). Deletion of the *ngr1* gene (*ngr1*$^{-/-}$) mice potentiated an upregulation in the numbers of NgR3^{+} T- and B-cell populations (n=5; *t* test ***p<0.05 and

**$p<0.01$ compared to controls, respectively). B220: B-cell marker; CD3e: T-cell marker. Bars represent mean ± SEM, *$p<0.05$, **$p<0.01$, ***$p<0.001$.

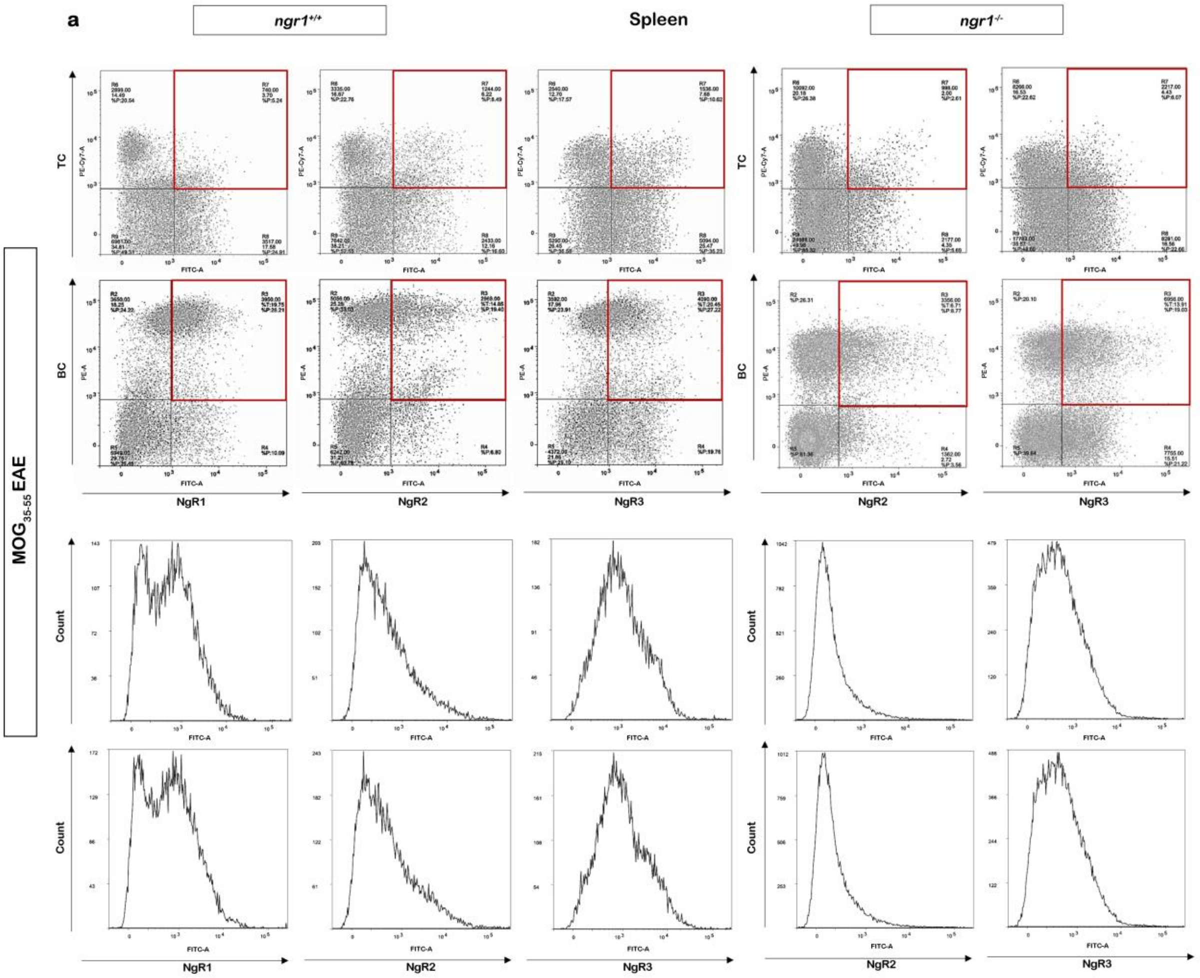
a
ngr1+/+
Spleen
ngr1-/-
MOG35-55 EAE
TC
BC
Count
NgR1
NgR2
NgR3
NgR2
NgR3

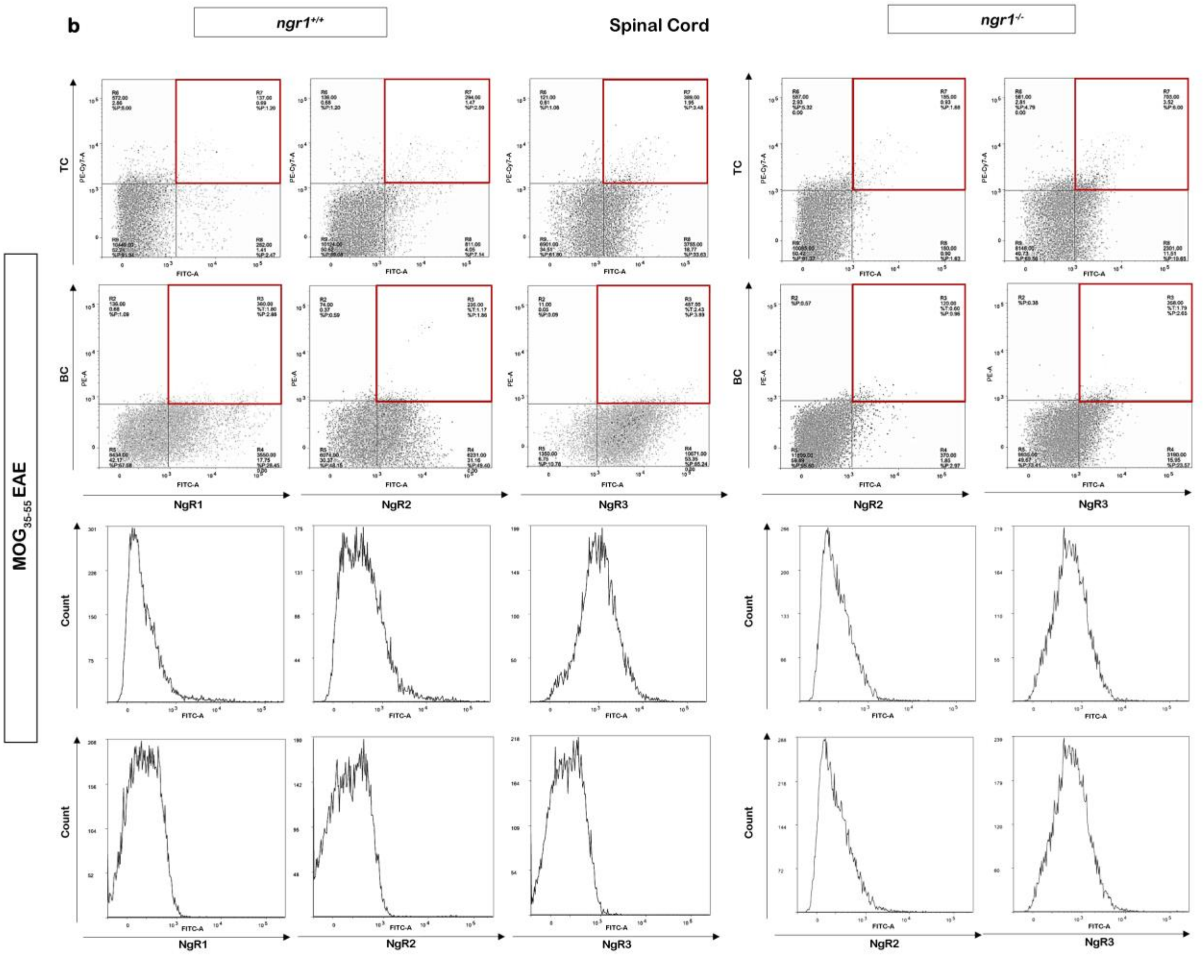
b
ngr1+/+
Spinal Cord
ngr1-/-
MOG35-55 EAE
TC
BC
Count
Count
PE-Cy7-A
PE-A
FITC-A
NgR1
NgR2
NgR3
NgR2
NgR3

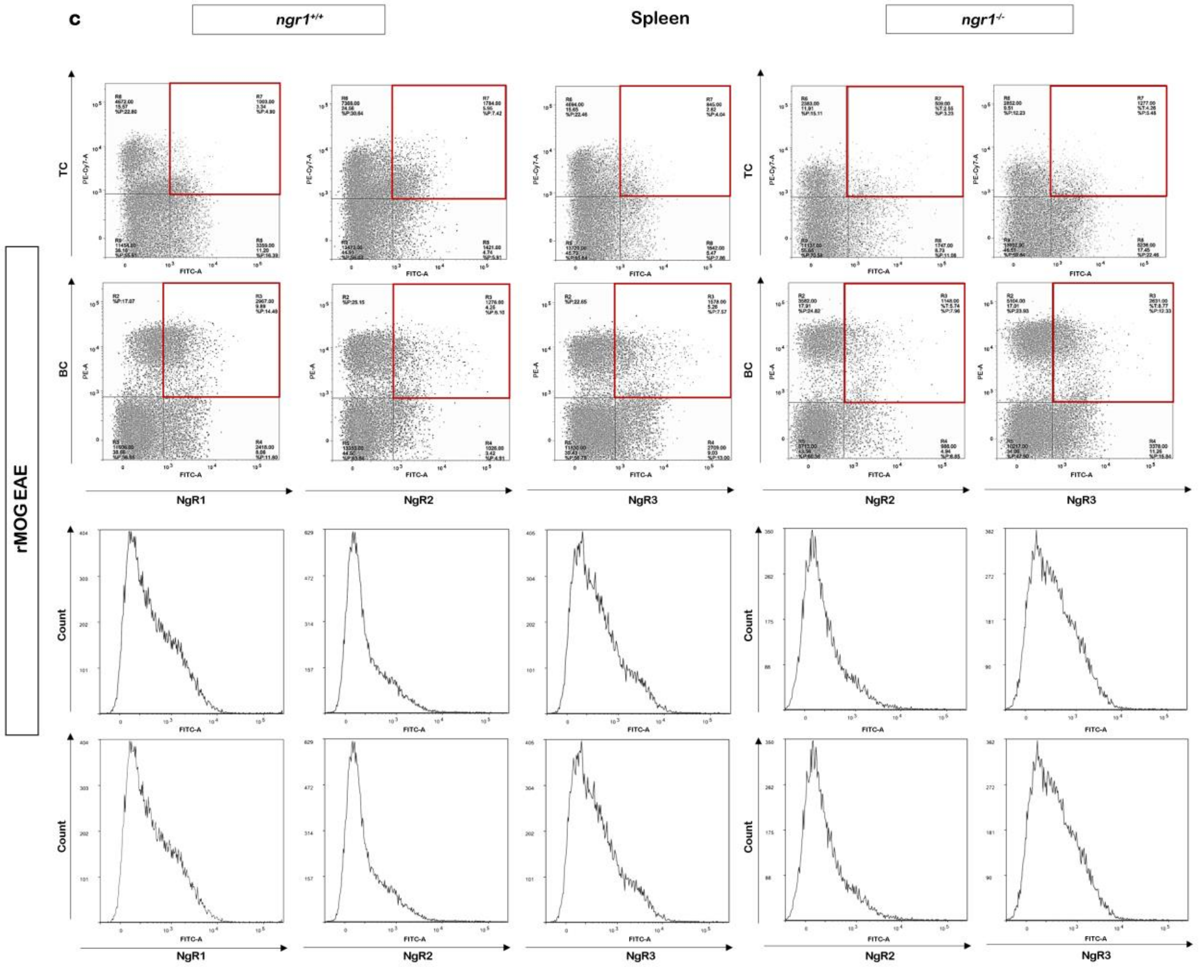
c
$ngr1^{+/+}$
Spleen
$ngr1^{-/-}$
rMOG EAE
TC
BC
PE-Cy7-A
PE-A
FITC-A
Count
NgR1
NgR2
NgR3
NgR2
NgR3

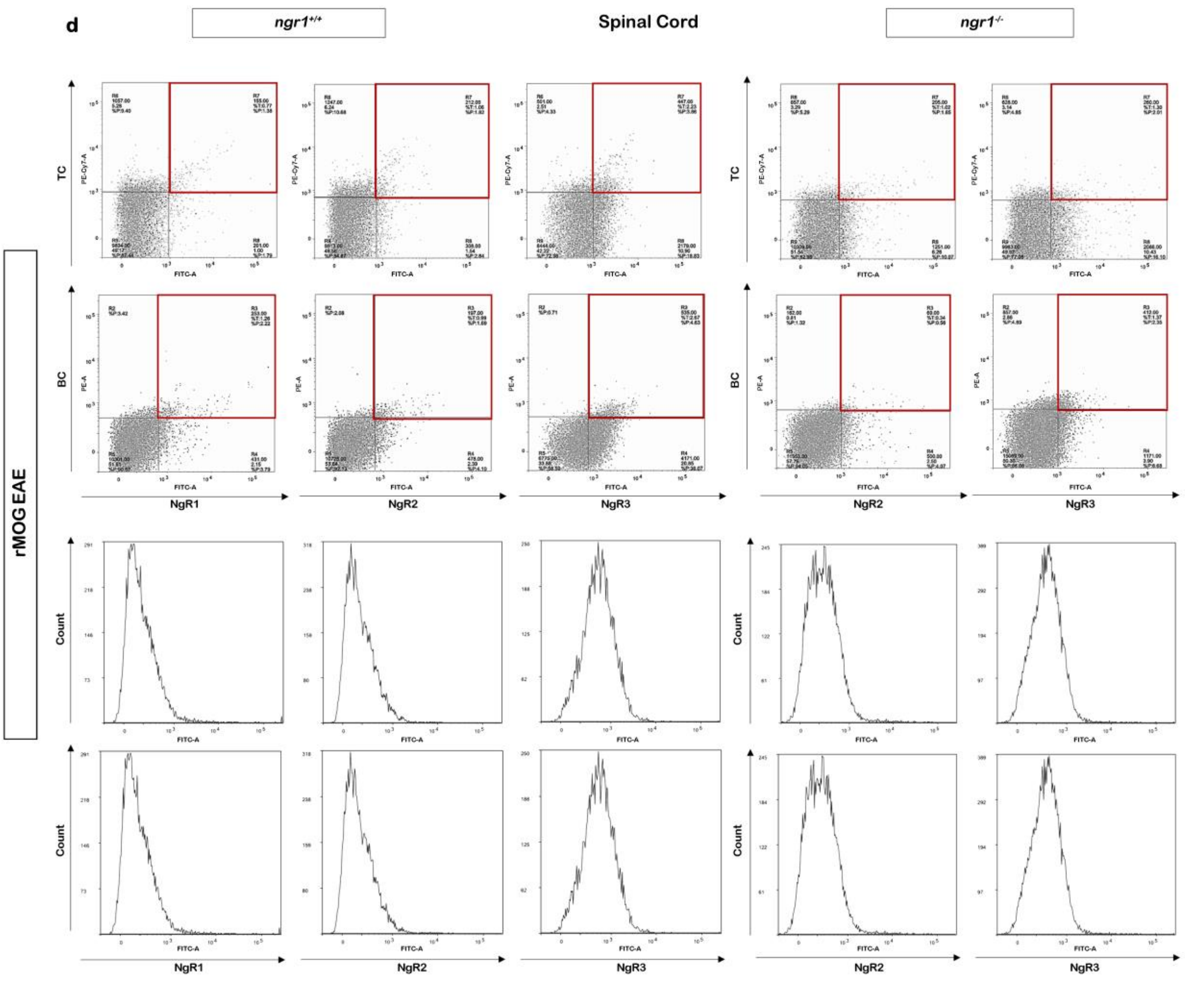

Additional file 2: Figure S2 The localisation of NgR1-3 on T and B-cells following $MOG_{35\text{-}55}$ or rMOG EAE-induction. **a** Representative flow cytometric plots performed from isolates of T- and B-cells from spleens of *ngr1*$^{+/+}$ and *ngr1*$^{-/-}$ mice following the induction of EAE with $MOG_{35\text{-}55}$ (clinical score 1, EAE onset of disease), that were co-labeled for NgR1, NgR2 and NgR3. **b** Representative flow cytometric plots of isolated T- and B-cells respectively, from the spinal cords of *ngr1*$^{+/+}$ and *ngr1*$^{-/-}$ mice following the induction of EAE with $MOG_{35\text{-}55}$ (clinical score 1, EAE onset of disease), that were co-labeled for NgR1, NgR2 and NgR3. **c** Representative flow cytometric plots of isolated T- and B-cells respectively, from the spleens of *ngr1*$^{+/+}$ and *ngr1*$^{-/-}$ mice following the induction of EAE with rMOG (clinical score 1, EAE onset of disease), that were co-labeled for NgR1, NgR2 and NgR3. **d** Representative flow cytometric plots of

isolated T- and B-cells respectively, from the spinal cords of *ngr1*$^{+/+}$ and *ngr1*$^{-/-}$ mice following the induction of EAE with rMOG (clinical score 1, EAE onset of disease), that were co-labeled for NgR1, NgR2 and NgR3.

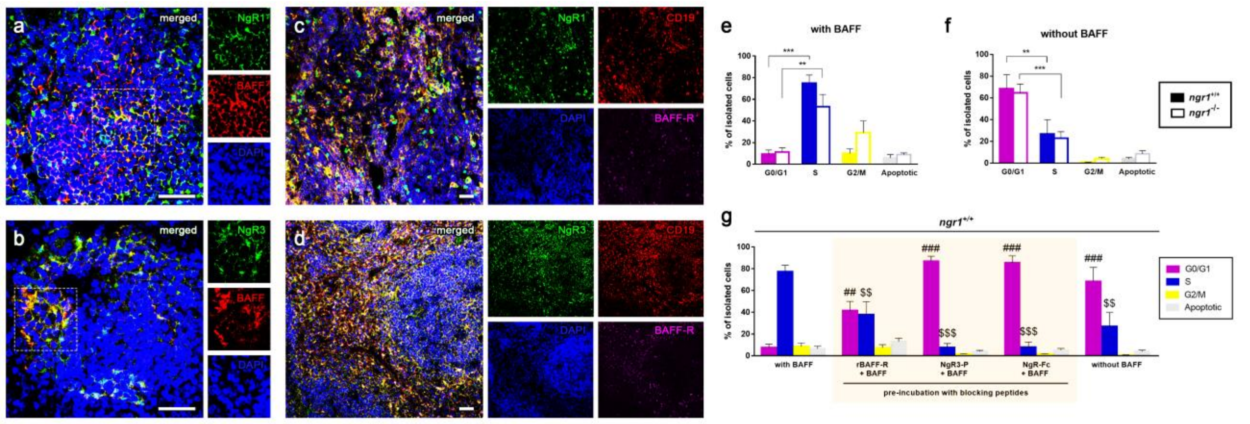

Additional file 3: Figure S3 BAFF plays a role in stimulating splenic B-cells that express NgR1 and NgR3 during the onset of EAE. **a, b** Clustered immune cell infiltrates that co-labeled either BAFF and NgR1 or, BAFF and NgR3, were found within the spleen of *ngr1*$^{+/+}$ EAE-induced mice at disease onset. **c, d** No NgR1^{+} or NgR3^{+} immune cells were detected in double-labeling experiments with BAFF-R within the white pulp of spleen from *ngr1*$^{+/+}$ mice; scale = 50 µm. **e, f** Double-labeled (B220^{+} NgR1^{+} and NgR3^{+}) B-cell populations were either stimulated with 50 ng/mL of recombinant BAFF (with BAFF) or left untreated (without BAFF). BAFF stimulation led to a significant increase in DNA synthesis (S phase compared to G0/G1 phase; n=10; *t* test ***p<0.001) of isolated cells from *ngr1*$^{+/+}$ mice while cells remained in the G0/G1 phase (n=10; *t* test *p<0.01) in the absence of BAFF. Isolated cells from *ngr1*$^{-/-}$ mice also exhibited significant differences between the G0/G1 and S phases in the BAFF treated cultures (n=8; *t* test *p<0.01) and those that were untreated (n=8; *t* test **p<0.001), highlighting the role of NgR3 in the absence of NgR1. **g** To identify NgR interactions with BAFF, rBAFF was blocked with an excess amount of either rBAFF-R, NgR1-Fc or NgR3-P. All three blocking peptides were efficacious at blocking the cell cycle-dependent BAFF activity when compared to the BAFF-treated cultures. Bars represent mean ± SEM; $^{\#}p$<0.05, $^{\#\#}p$<0.01, $^{\#\#\#}p$<0.001 comparisons with the G0/G1 phase of the BAFF-treated cultures, *t* test (with Bonferroni correction); $^{\$}p$<0.05, $^{\$\$}p$<0.01, $^{\$\$\$}p$<0.001 comparisons with the S phase of the BAFF-treated cultures, *t* test (with Bonferroni correction).

Supplementary Immunoblot data

We provide the full-length versions of all blots to be included in the Supplementary file, as per journal policy.

For Figure 2a

We ran the control and the disease onset time-point juxtaposed in every single gel. Lanes 1-2 represent NgR1, lanes 3-4 is the β-actin and 5-6 is the NgR3 specific antibody.

A. Spleen ngr1 WT

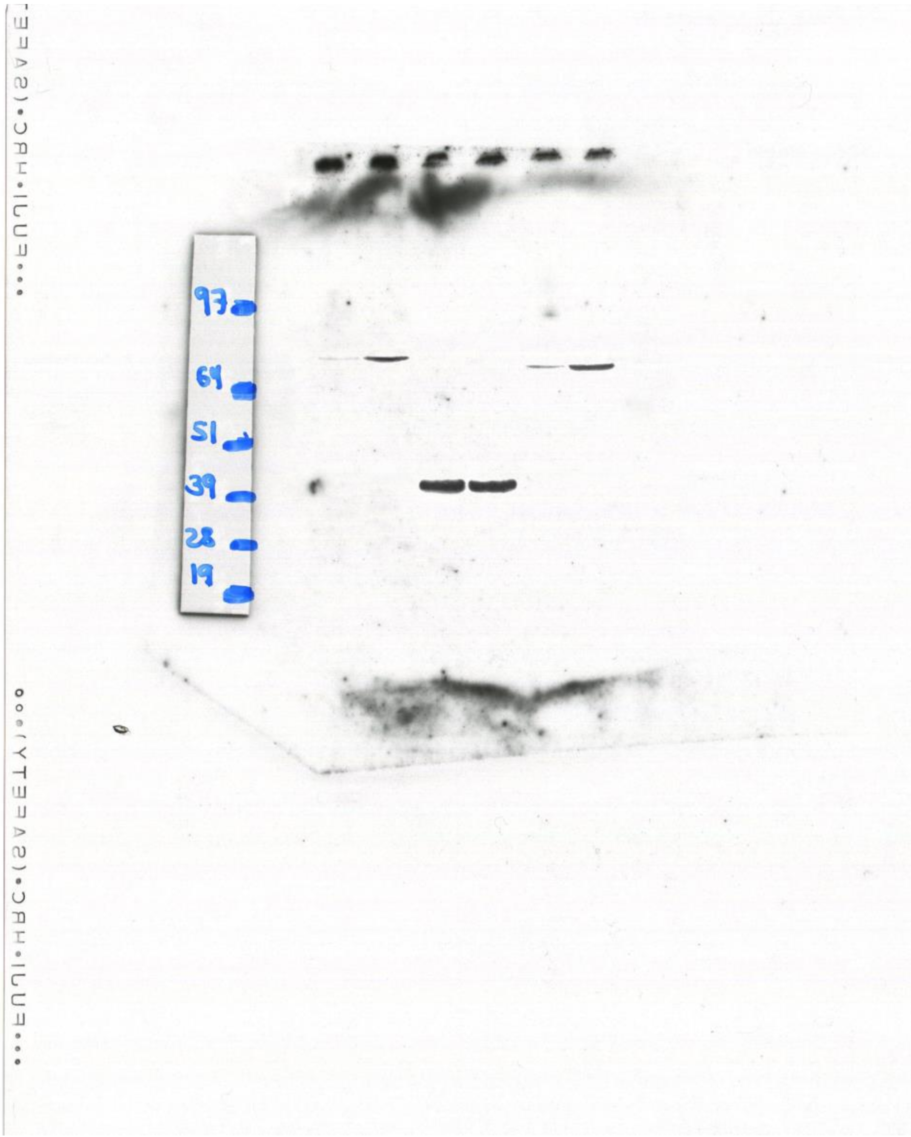

B. Spleen ngr1 KO

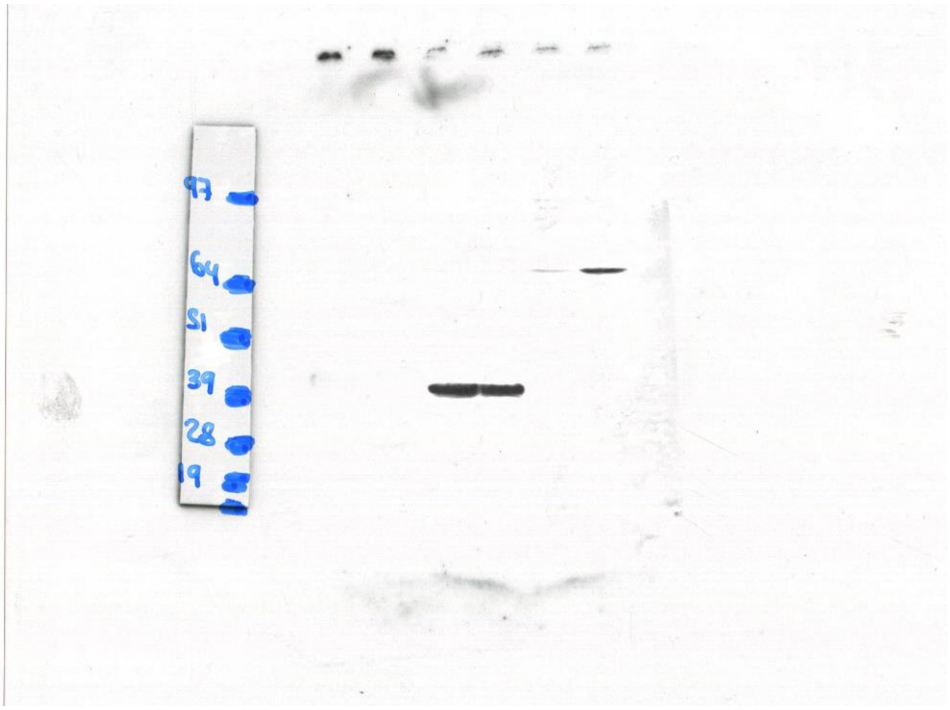

C. Spinal cord ngr1 WT

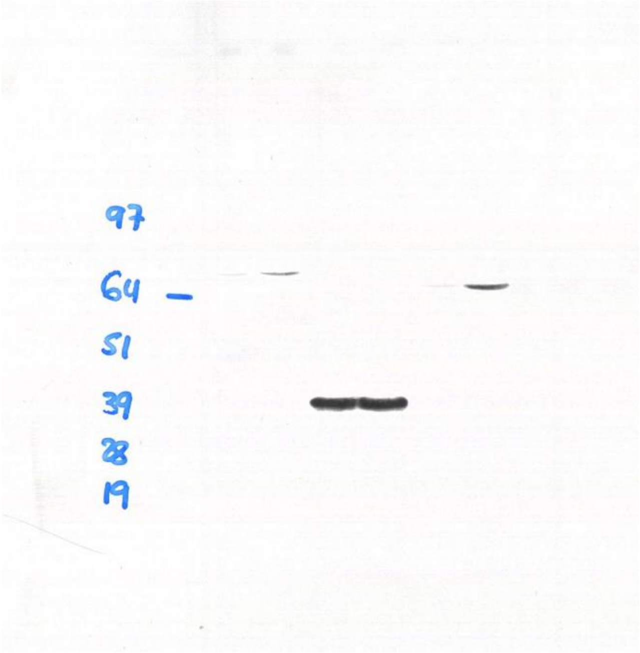

D. Spinal cord ngr1 KO

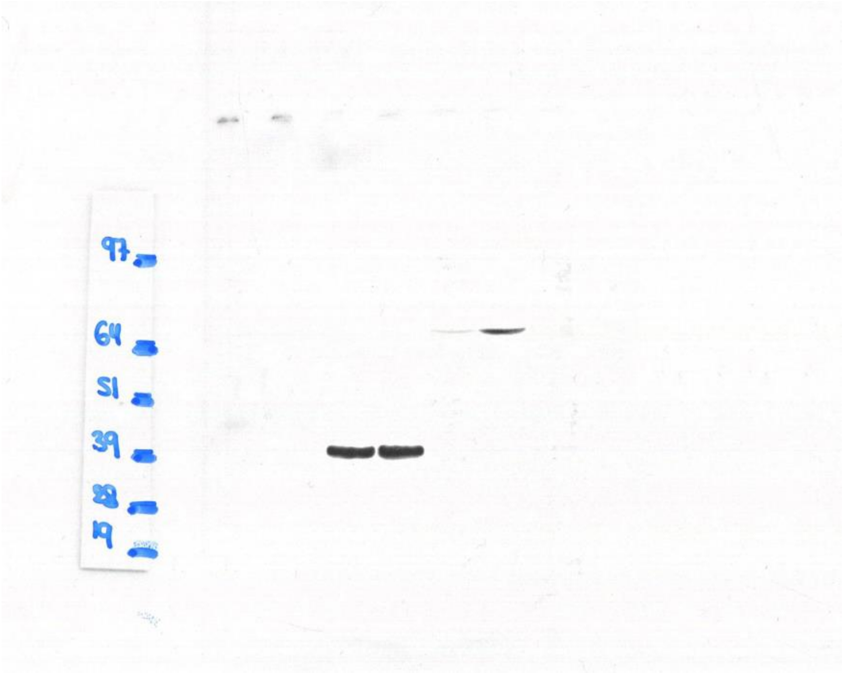

(As a panel)

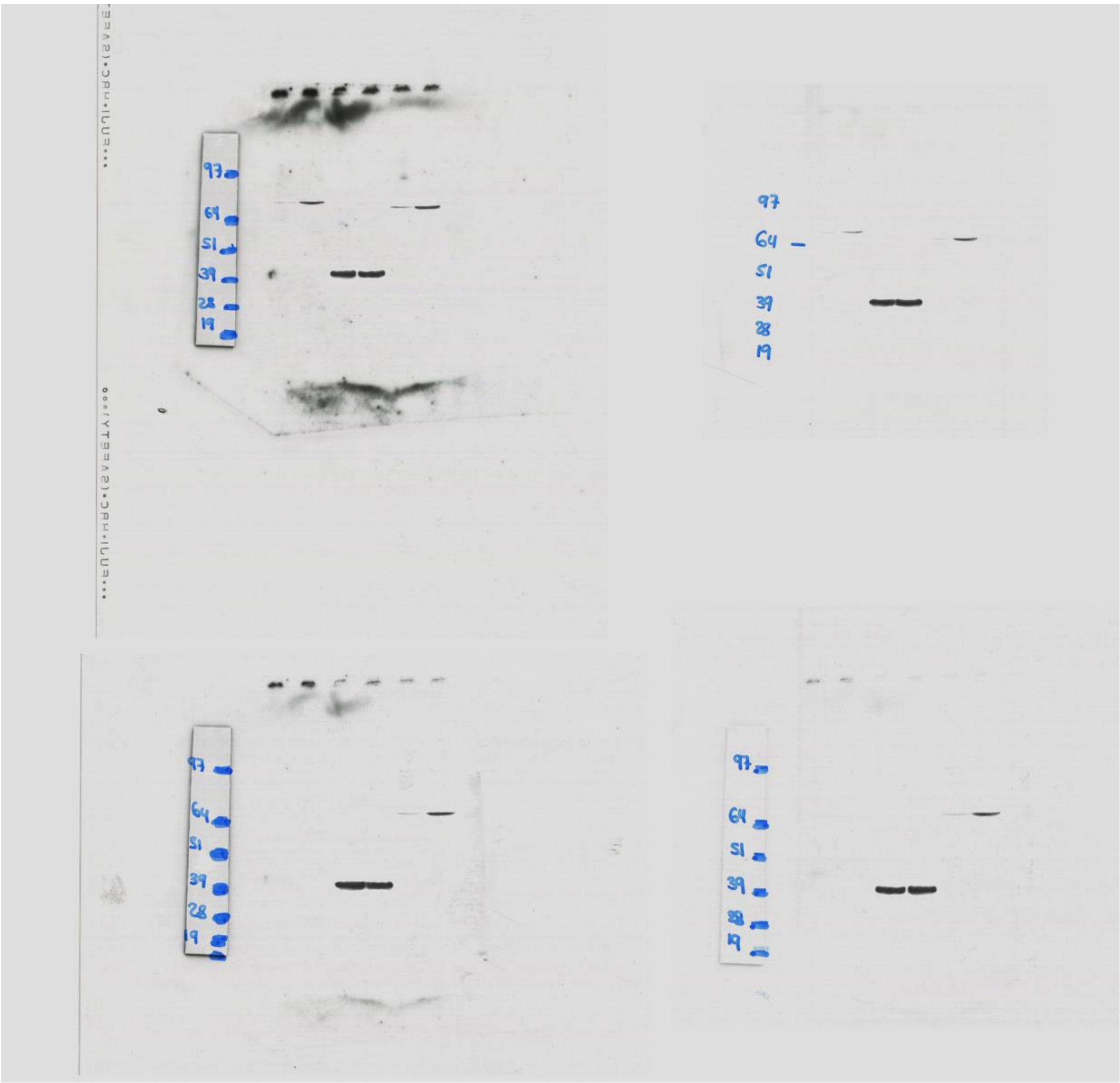

For Figure 5c

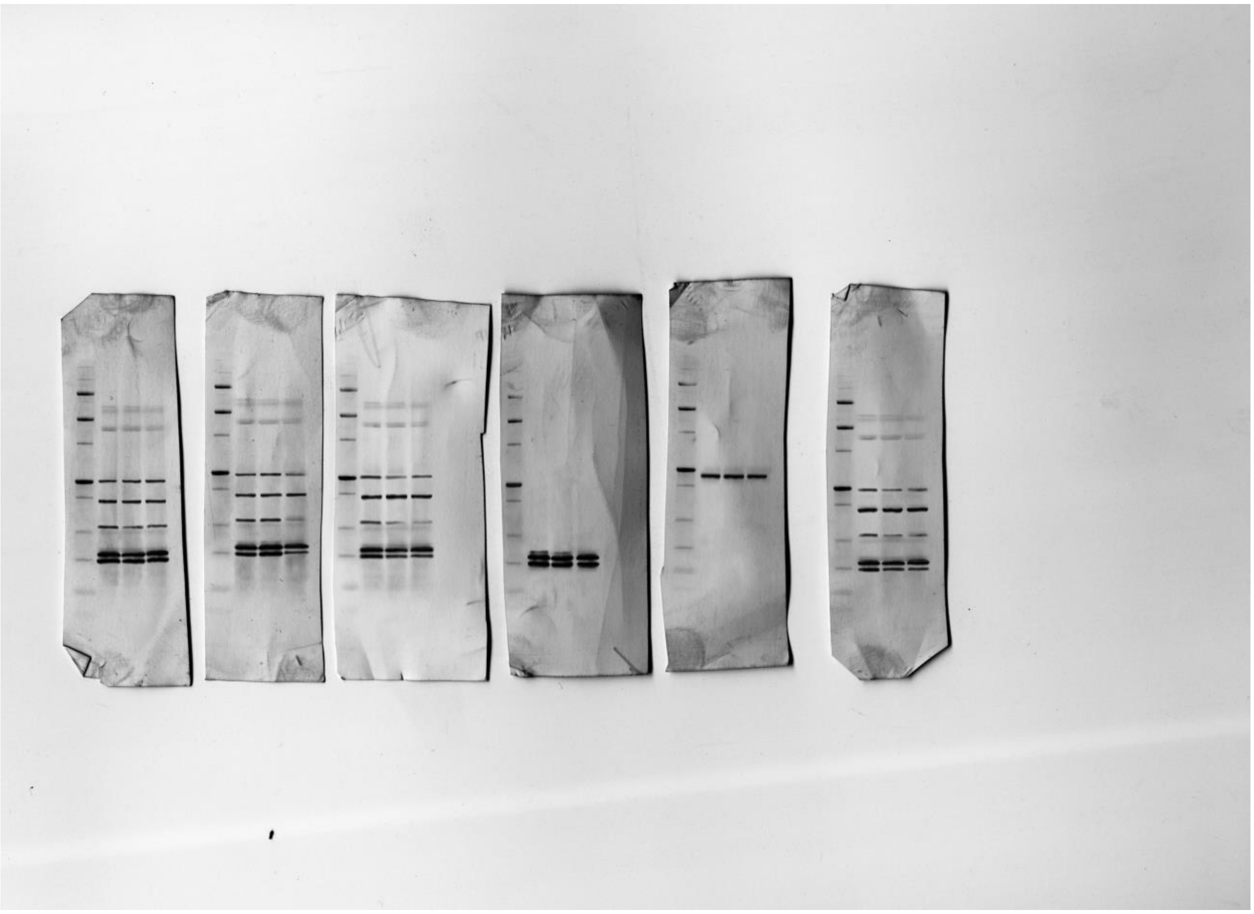

(from Left to Right)

Blot 1: Spleen isolates - NgR1^{+} B-cells

Blot 2: Spleen isolates - NgR3^{+} B-cells

Blot 3: Spinal cord isolates – NgR1^{+} B-cells

Blot 4: controls - MBP

Blot 5: controls – β-actin

Blot 6: Spinal cord isolates – NgR3^{+} B-cells

For Figure 5c

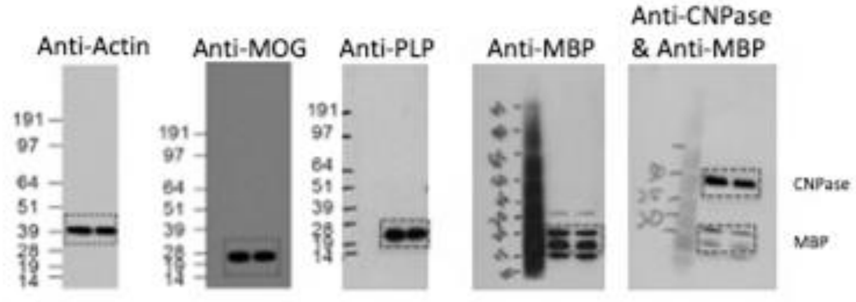

Figure S4. The full immunoblots of Controls for Figs 5C

The full immunoblot membranes of the representative controls using commercial antibodies for all CNS myelin proteins depicted in Figure 5C. Dotted lines indicate regions of the blots used in each figure for Actin, MOG, PLP, MBP and CNPase, respectively.
