# Supplementary material for: B-cells expressing NgR1 and NgR3 are localized to EAE-induced inflammatory infiltrates and are stimulated by BAFF
Source: Sci Rep. 2021 Feb 3;11:2890. doi: 10.1038/s41598-021-82346-6 (PMC7858582; doi:10.1038/s41598-021-82346-6)
Supplement: Supplementary file 2 — Supplementary Table S1. [file 41598_2021_82346_MOESM2_ESM.docx]

**SUPPLEMENTARY TABLE 1**

| ***Antibody*** | ***Company*** | ***Catalogue #*** | ***Technique*** | ***Dilution*** |
| --- | --- | --- | --- | --- |
| Anti-BAFF Receptor Biotinylated | R&D Systems | BAF1537 | IHC | 1 in 200 |
| Anti-BLyS/BAFF | Novus Biologicals | NB100-56310 | IHC | 1 in 100 |
| Anti-BrdU | BD Biosci | 552598 | ICC | Kit |
| Anti-CD268/BAFF Receptor | EBiosci | 14-9117-82 | IHC | 1 in 100 |
| Anti-CD3e-PE-Cy7 | EBiosci | 25-0031-81 | Flow | 1 in 100 |
| Anti-CD19-AlexFluor® 594 | BioLegend | 115552 | IHC | 1 in 100 |
| Anti-CD20 | BioLegend | 152104 | IHC | 1 in 100 |
| Anti-CD45/B220 | Bioss | bs-0522R | IHC & ICC | 1 in 200 |
| Anti-CD45/B220-PE | BD Pharmingen | 553090 | Flow | 1 in 200 |
| Anti-CNPase | Millipore | MAB326R | IHC | 1 in 200 |
| Anti-IgD-AP | Southern Biotech | 405714 | ELISA | 1 in 200 |
| Anti-IgD-APC | BioLegend | 405714 | Flow | 1 in 200 |
| Anti-IgG-AP | Southern Biotech | 1070-04 | ELISA | 1 in 2000 |
| Anti-IgG-Biotin | EBiosci | 13-4013-85 | Flow | 1 in 200 |
| Anti-IgM-AP | Southern Biotech | 0101-01 | ELISA | 1 in 2000 |
| Anti-IgM-PerCP/Cy5.5 | BioLegend | 406512 | Flow | 1 in 400 |
| Anti-Ki67-AlexaFluor® 450 | EBiosci | 48-5698-80 | Flow | 1 in 200 |
| Anti-MBP | Sapphire Biosci | AB40390 | IHC & WB | 1 in 10000 |
| Anti-MOG | Millipore | 2116701 | IHC | 1 in 1000 |
| Anti-Mouse antibody-HRP | Merck Millipore | 402335 | WB | 1 in 10000 |
| Anti-NgR1 | R&D Systems | AF1440 | IHC, ICC & WB | 1 in 100 |
| Anti-NgR1 | Millipore | AB15138 | IHC, ICC & WB | 1 in 100 |
| Anti-NgR2 | Santa Cruz Biotech | sc-168752 | Flow & IHC | 1 in 100 |
| Anti-NgR3 | Santa Cruz Biotech | sc-165108 | Flow, IHC, ICC & WB | 1 in 100 |
| Anti-NogoA | Millipore | AB5888 | IHC & WB |  |
| Anti-PLP Rabbit Polyclonal | Abcam | 120-28486 | IHC & WB | 1 in 1000 |
| Anti-Rabbit antibody-HRP | Merck Millipore | 12-348 | WB | 1 in 1000 |
| Anti-Rat antibody-HRP | Merck Millipore | AP136P | WB | 1 in 1000 |
| Anti-Streptavadin APC | EBiosci | 17-4317-82 | Flow | 1 in 300 |
| Donkey anti-Goat 488 | Life Technologies | A11055 | IHC & Flow | 1 in 200 |
| Donkey anti-Goat 555 | Life Technologies | A21432 | IHC & Flow | 1 in 200 |
| Goat Anti-Rabbit IgG-Alexa Fluro® 555 | Life Technologies | A21428 | IHC & Flow | 1 in 200 |
| Goat Anti-Rabbit IgG-AlexaFluro® 488 | Life Technologies | A11008 | IHC & Flow | 1 in 200 |
| Goat Anti-Rat IgG-Alexa Fluro® 488 | Life Technologies | A11006 | IHC & Flow | 1 in 200 |
| Goat Anti-Rat Alexa Fluor®647 | Life Technologies | A21247 | IHC | 1 in 200 |
| Goat Anti-Rat IgG-Alexa Fluro® 555 | Life Technologies | A21434 | IHC & Flow | 1 in 200 |
| Goat-Anti-Mouse IgG1-AP | Southern Biotech | 1071-04 | ELISA | 1 in 2000 |
| Hamster IgG isotype control-PE-Cy7 | EBiosci | 25-4888-81 | Flow | 1 in 200 |
| Rat IgG 2b kappa isotype Control-PE | BD Pharmingen | 559478 | Flow | 1 in 200 |
| Streptavidin | BD Pharmingen | 551419 | IHC | 1 in 200 |

**Legend:** A list of all commercially-available species-specific antibodies that were utilised in the outlined experiments, executed within this original study. All catalogue numbers for these antibodies, the methods where they were included, and dilutions used throughout the experiments are listed.
